# Transcript shortening via alternative polyadenylation promotes gene expression during fracture healing

**DOI:** 10.1038/s41413-022-00236-7

**Published:** 2023-01-03

**Authors:** Deepak Kumar Khajuria, Irena Nowak, Ming Leung, Vengadeshprabhu Karuppagounder, Yuka Imamura, Christopher C. Norbury, Fadia Kamal, Reyad A. Elbarbary

**Affiliations:** 1 Department of Orthopaedics and Rehabilitation, The Pennsylvania State University College of Medicine, Hershey, PA 17033 USA; 2 Center for Orthopaedic Research and Translational Science (CORTS), The Pennsylvania State University College of Medicine, Hershey, PA 17033 USA; 3 Institute for Personalized Medicine, Penn State College of Medicine, Hershey, PA 17033 USA; 4 Department of Pharmacology, The Pennsylvania State University College of Medicine, Hershey, PA 17033 USA; 5 Department of Microbiology and Immunology, The Pennsylvania State University College of Medicine, Hershey, PA 17033 USA; 6 Department of Biochemistry and Molecular Biology, The Pennsylvania State University College of Medicine, Hershey, PA 17033 USA; 7 Center for RNA Molecular Biology, Pennsylvania State University, University Park, PA 16802 USA

**Keywords:** Bone, Bone quality and biomechanics

## Abstract

Maturation of the 3′ end of almost all eukaryotic messenger RNAs (mRNAs) requires cleavage and polyadenylation. Most mammalian mRNAs are polyadenylated at different sites within the last exon, generating alternative polyadenylation (APA) isoforms that have the same coding region but distinct 3′ untranslated regions (UTRs). The 3′UTR contains motifs that regulate mRNA metabolism; thus, changing the 3′UTR length via APA can significantly affect gene expression. Endochondral ossification is a central process in bone healing, but the impact of APA on gene expression during this process is unknown. Here, we report the widespread occurrence of APA, which impacts multiple pathways that are known to participate in bone healing. Importantly, the progression of endochondral ossification involves global 3′UTR shortening, which is coupled with an increased abundance of shortened transcripts relative to other transcripts; these results highlight the role of APA in promoting gene expression during endochondral bone formation. Our mechanistic studies of transcripts that undergo APA in the fracture callus revealed an intricate regulatory network in which APA enhances the expression of the collagen, type I, alpha 1 (Col1a1) and Col1a2 genes, which encode the 2 subunits of the abundantly expressed protein collagen 1. APA exerts this effect by shortening the 3′UTRs of the Col1a1 and Col1a2 mRNAs, thus removing the binding sites of miR-29a-3p, which would otherwise strongly promote the degradation of both transcripts. Taken together, our study is the first to characterize the crucial roles of APA in regulating the 3′UTR landscape and modulating gene expression during fracture healing.

## Introduction

Endochondral ossification is the process by which long bones form during development, and it is the most common process by which adult bones heal.^1–3^ Most cortical bone fracture healing occurs via the initial formation of a fibrocartilaginous (soft) callus that is filled with proliferating chondrocytes; at later stages, these proliferating chondrocytes terminally differentiate into hypertrophic chondrocytes and secrete extracellular matrix components, including type I and type 10 collagen, that mediate the gradual mineralization of the soft callus and hardening of the fracture gap.^1–3^ This facilitates the invasion of new blood vessels and subsequent replacement of chondrocytes with newly formed woven bone via the endochondral ossification process.^1–3^

The 3′ end of most eukaryotic messenger RNAs (mRNAs) is formed through the cotranscriptional recognition of a specific sequence motif called the polyadenylation signal (PA), followed by endonucleolytic cleavage and the addition of a polyadenosine (poly A) tail at the cleavage and polyadenylation site (CPS).^4–7^ More than 70% of mammalian genes have multiple PAs, the utilization of which results in the expression of alternative polyadenylation (APA) isoforms.^7,8^ Most PAs are localized within the last exon, so APA isoforms have the same coding sequence (CDS) but differ in 3′ untranslated region (UTR) length.^9^ The 3′ UTR contains several sequence and structure motifs, including microRNA (miRNA) binding sites, that regulate mRNA metabolism at several levels.^7,8,10^ miRNAs are short ~22-nucleotide RNAs that direct the posttranscriptional repression of most mammalian genes by inhibiting mRNA translation and/or promoting mRNA decay.^11,12^ Accordingly, the removal of miRNA binding sites and other important regulatory motifs from the 3′ UTR via APA can significantly impact mRNA translation, stability, and/or localization.^7,8,10^ Although less probable, PAs might also be present within introns, leading to APA isoforms with different CDSs.^13,14^ APA is regulated during different biological processes. For example, rapidly proliferating cells exhibit widespread shortening of 3′ UTRs, which is in stark contrast to the general trend of 3′ UTR lengthening that occurs in differentiating cells.^8,10^

The increased interest in APA and its substantial roles in the posttranscriptional regulation of gene expression has prompted the development of novel bioinformatics tools to globally identify and quantify APA isoforms in RNA-sequencing (RNA-seq) data. One powerful tool is Dynamic Analyses of Alternative PolyAdenylation from RNA-Seq (DaPars), which quantifies dynamic APA events via the analysis of localized changes in RNA-seq density near the 3′ ends of mRNAs.^15^ DaPars has been adopted by many databases and is used to analyze changes in the abundance of APA isoforms under different biological conditions; for example, DaPars has been used to analyze 3′ UTR shortening in samples from The Cancer Genome Atlas.^8,16^ The limitations of DaPars include that it can only be used with two-group, but not one-group, datasets. In addition, the coverage of RNA-seq reads around tandem poly(A) sites is not uniform, which increases the rate of false positives in the assessment of PA usage.^8^ To address these limitations, other bioinformatics tools have been developed, including the Identification of Novel alternative PolyAdenylation Sites (InPAS) algorithm. InPAS is based on and was developed from DaPars; however, it adjusts cleavage sites by utilizing annotation packages to correct 3′ UTR ranges and prevent false CPS detection.^17,18^ Recently, the APAlyzer bioinformatic toolkit has also been developed to overcome the intrinsic limitation of de novo CPS prediction by utilizing the comprehensive CPS collection in the PolyA_DB database.^19–21^ The APAlyzer also examines APA events in all genic regions, including 3′ UTRs and introns.^18,19^

Although the occurrence of APA and its roles in regulating gene expression have been identified in several tissues and disease conditions,^8,15,18,22^ these events are understudied in musculoskeletal tissues and uncharacterized in bone regeneration. Here, we analyzed APA events in the healing callus for the first time and elucidated their roles in the posttranscriptional regulation of gene expression during endochondral ossification.

## Results

### Replacement of the cartilaginous callus with woven bone is associated with increased cell proliferation and immune cell infiltration

**Fig. 1:**
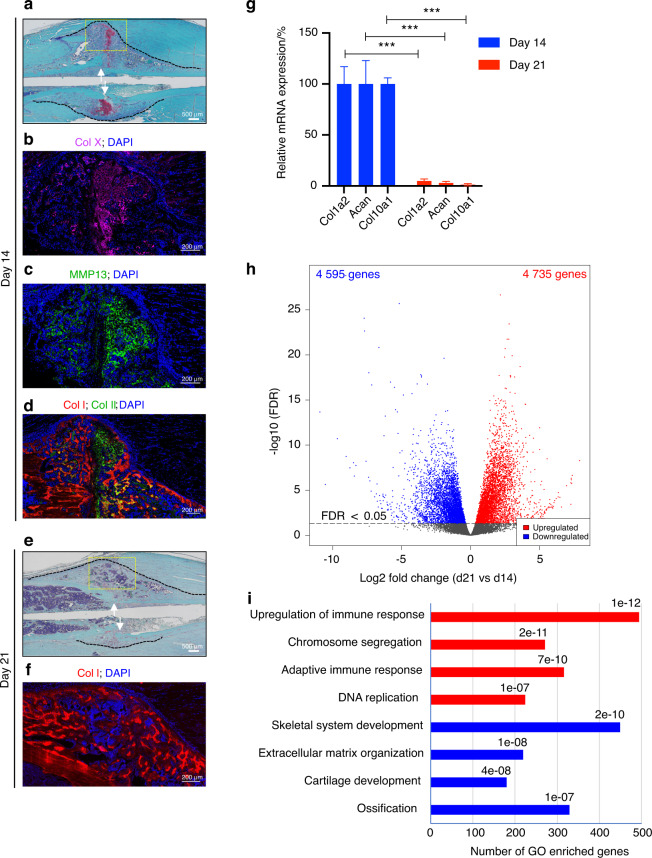
Cells with higher proliferation capacity populate the callus as the cartilaginous callus that bridges the fracture gap is replaced by woven bone. **a**–**d** Staining of d14 calli. **a** Safranin O/fast green staining. The cartilaginous extracellular matrix of the soft callus was stained reddish orange. The white arrows point to the fracture line. The dashed black line outlines the callus. The scale bar = 500 μm. IF staining of Col X (purple) (**b**) and MMP13 (green) (**c**). Both proteins are secreted by hypertrophic chondrocytes. **d** IF costaining of Col I (red) and Col II (green). Areas where both proteins are expressed (yellow) surround mineralized chondrocytes. IF images shown in **b**–**d** were captured in the region indicated by the yellow box in **a**. **e** As in **a** except that d21 callus samples were stained. The absence of a reddish orange stain indicates resorption of the cartilaginous callus. **f** As in **d** except that d21 callus samples were stained. The image was captured in the region indicated by the yellow box in **e**. The trabecular structure of woven bone bridging the fracture gap is obvious. In all the IF images, DAPI stains nuclei (blue), and the scale bar = 200 μm. All the images are representative of five callus tissues. **g** RT‒qPCR quantification of the indicated transcripts in the RNA that was purified from d14 and d21 callus tissues. Both Col1a2 and Aggrecan (Acan) are chondrocyte markers, while Col10a1 is a hypertrophic chondrocyte marker. The expression level of each transcript was normalized to that of β-actin mRNA, and the normalized level on d14 was defined as 100. *n* = 3. The bar graphs present the average ± SEM. ***P < 0.001 using unpaired Student’s *t* test. **h** A volcano plot of RNA-seq data displaying the fold change in gene expression values on d21 relative to d14 (Table S1). Significantly differentially expressed genes (FDR < 0.05) are highlighted in red and blue to indicate upregulated and downregulated genes, respectively. RNA-seq data were generated from three biological replicates at each timepoint. **i** GO analysis of significantly differentially expressed genes shown in **h**. Representative examples of functional categories enriched by upregulated (red) or downregulated (blue) genes are shown, and the adjusted *P* value (*P*_adj_) for each pathway is given (see also Figs. S5 and S6a, b and Tables S2 and S3 for comprehensive lists of significantly enriched functional categories)

We performed tibial diaphyseal osteotomy and stabilized the fracture using an intramedullary nail, this is an established model of secondary fracture healing that proceeds through the endochondral ossification process.^1–3^ As we and others have reported for this model,^23–25^ at the end of the inflammatory phase and the early repair phase, i.e., at Day 7 (d7) postfracture, the fracture gap was filled with and surrounded by a fibrocartilaginous (soft) callus (Fig. S1). The soft callus increased in size as the repair phase advanced to d10 (Fig. S1).^23^ As healing further proceeded to d14, the densely packed chondrocytes that filled the soft callus progressed into a hypertrophic state (Figs. 1a and S2a, b) and secreted the hypertrophy markers type 10 collagen (Col X) (Fig. 1b) and matrix metallopeptidase 13 (MMP13) (Fig. 1c). Additionally, a subset of soft-callus chondrocytes became terminally hypertrophic, as shown by the secretion of type I collagen (Col I) (Figs. 1d and S2c, d), which is an essential protein for soft-callus mineralization. At the 3 timepoints (i.e., d7 to d14), the callus areas distal to the fracture gap were filled with newly formed bone that was interwoven with bone marrow (Figs. S1 and S2b, c, f). As expected, immune cells were enriched in the bone marrow that populated the woven-bone regions, while fewer immune cells were observed in the soft-callus area (Fig. S2e, g). As healing further progressed from d14 to d21, endochondral ossification mediated the replacement of the cartilaginous callus with newly formed woven bone, which was accompanied by the re-establishment of bone marrow elements, including immune cell populations (Figs. 1e, f and S3a–f). The quantitative measurement of gene expression using reverse transcriptase combined with quantitative polymerase chain reaction (RT‒qPCR) corroborated our histological analysis and demonstrated very low expression of chondrocyte and hypertrophic chondrocyte markers at d21 (Fig. 1g); these results indicated the nearly complete resorption of the cartilaginous callus.

To gain insight into the biological processes involved in the calcification and resorption of the cartilaginous callus as well as the formation of woven bone, we performed RNA-seq using total RNA that was isolated from callus tissues on d14 and d21. We identified 9 330 differentially expressed genes (*P*_adj_ < 0.05; DESeq2^26^) at d21 relative to d14 (Figs. 1h and S4 and Table S1). As expected, the expression of genes involved in cartilage development and ossification was downregulated at d21 compared to d14 (Gene Ontology (GO) analysis;^27,28^ Figs. 1i and S5 and S6a, b and Tables S2 and S3). On the other hand, d21 showed the upregulation of genes in several pathways that are involved in immune cell responses (Figs. 1i and S5 and S6a, b and Tables S2 and S3), which was consistent with the re-establishment of the bone marrow population and the replacement of the dense fibrocartilaginous callus, which has relatively low immune cell infiltration, with the marrow-infiltrated woven bone (Figs. S2 and S3). RT‒qPCR analysis of the relative expression of the immune cell marker CD45 between Days 7 and 21 demonstrated the lowest CD45 expression levels on Days 10 and 14 (Fig. S6c), which are the 2 timepoints that are distant from the initial immune response and are characterized by the largest soft callus areas. On the other hand, the expression level of CD45 was highest on d21 among the 4 analyzed timepoints, and it was increased by ∼7 fold on d21 relative to d14 (Fig. S6c), which was consistent with the RNA-seq data (Table S1) and GO analysis (Figs. 1i and S5 and S6a, b and Tables S2 and S3). Importantly, GO analysis also showed that cell division, DNA replication, and chromosome segregation were among the most upregulated pathways on d21 relative to d14 (Figs. 1i and S5 and S6a, b and Tables S2 and S3). Accordingly, as endochondral ossification proceeds, rapidly proliferating cells, including immune cells, populate the callus to replace cells with lower proliferation capacities. These observations are important to our studies, as rapidly proliferating cells are reported to exhibit global 3′ UTR shortening^8,10^ (see below).

### The transcriptome of the healing callus exhibits widespread utilization of alternative PAs

**Fig. 2:**
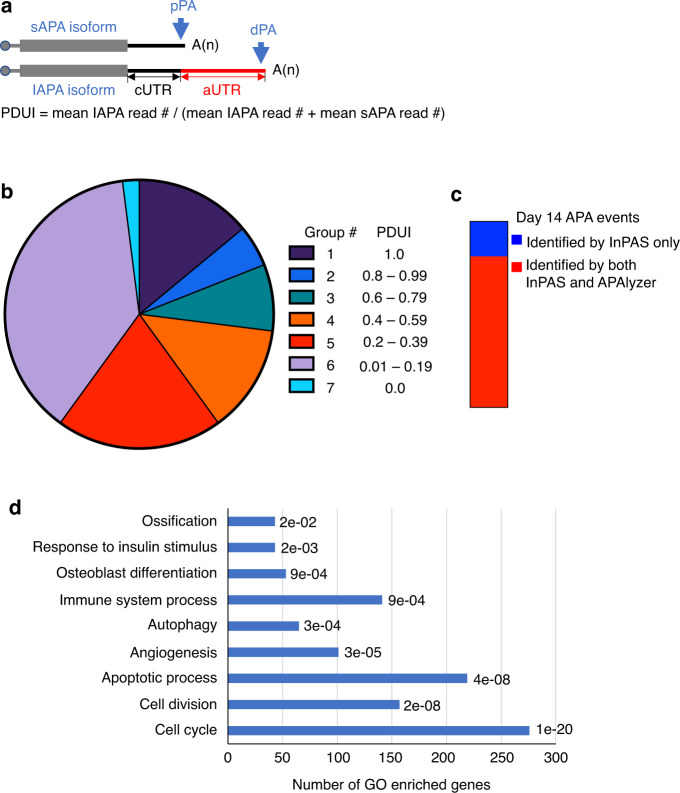
APA is commonly found in transcripts that are expressed in the callus on day 14 postfracture. **a** Schematic of the short APA (sAPA) and long APA (lAPA) isoforms of the same gene. Utilization of the proximal polyadenylation site (pPA) confines the size of the 3′ UTR to the constitutive UTR (cUTR), generating the sAPA isoform. On the other hand, utilization of the distal PA (dPA) results in the inclusion of the alternative UTR (aUTR) of the 3′ UTR, generating the lAPA isoform. The InPAS-based calculation of PDUI values is provided. **b** Pie chart presentation of the distribution of the indicated 7 PDUI groups in the transcriptome of the d14 callus. **c** Presentation of the overlap between the APA events in the transcriptome of the d14 callus that were identified by InPAS and APAlyzer. Events identified by both programs are shown in red; events identified by InPAS alone are shown in blue. **d** GO analysis of APA events in d14 calli. Significantly enriched functional categories are shown, and the false discovery rate (FDR) of enrichment for each pathway is given (comprehensive lists of significantly enriched functional categories are provided in Fig. S7 and Table S6)

When the last exon of a gene contains multiple PAs (Fig. 2a), utilization of the distal PA (dPA) during mRNA maturation results in the incorporation of the alternative UTR (aUTR) sequences, generating an APA isoform with a longer 3′ UTR (long APA isoform, or “lAPA”) (Fig. 2a). On the other hand, utilization of the proximal PA (pPA) removes the aUTR region and limits the size of the 3′ UTR to the constitutive UTR (cUTR), generating a short APA isoform (sAPA) (Fig. 2a). To study the extent of APA in the healing callus, we first performed transcriptomic analysis of the APA isoforms in the callus on d14 using the DaPars-based InPAS algorithm.^17,18^ Quantification of the relative expression of APA isoforms is based on calculating the Percent of Distal Utilization Index (PDUI) (Fig. 2a), which indicates the fraction of each analyzed transcript that is expressed as a lAPA isoform (Fig. 2a). The results indicated that ∼14% of the analyzed genes exclusively utilized the dPA (PDUI = 1; Group 1 in Fig. 2b; Table S4), while ∼2% exclusively utilized the pPA (PDUI = 0, Group 7 in Fig. 2b; Table S4). Importantly, ∼84% of the analyzed genes utilized both the dPA and pPA to variable extents (Groups 2-6 in Fig. 2b; Table S4). Hereafter, this group will be called APA genes/events. The same analysis was performed using APAlyzer, which utilizes a different approach to quantify APA isoform expression, and this analysis yielded comparable data, as APA events were identified in ∼78% of the genes, which showed ∼81% overlap with the APA events identified by InPAS (Fig. 2c and Table S5). Importantly, in ∼50% of the APA genes, the minor APA isoform constituted ≥20% of the total gene reads (Fig. 2b). GO analysis^29^ of the APA genes indicated significant enrichment in a wide range of biological pathways that play essential roles in fracture healing (Figs. 2d and S7 and Table S6). In fact, a large number of transcripts that are known to play roles in ossification, angiogenesis, osteoblast differentiation, endochondral ossification, and extracellular matrix deposition generated a PDUI of 40%–50%, suggesting substantial utilization of both the pPA and dPA (Table S7). These genes included the bone matrix components collagen, type I, alpha 1 (Col1a1) and alpha 2 (Col1a2); the angiogenic factor vascular endothelial growth factor A (Vegfa); the osteoclast marker tartrate resistant acid phosphatase 5 (Acp5; also known as TRAP); and the bone/cartilage development inducer bone morphogenic protein 5 (Bmp5) (Table S7). These results indicate that thousands of genes are expressed as multiple APA isoforms in the callus and suggest critical roles of APA in bone regeneration.

### Utilization of the pPA generates shortened 3′ UTRs in the Col1a1 and Col1a2 transcripts

**Fig. 3:**
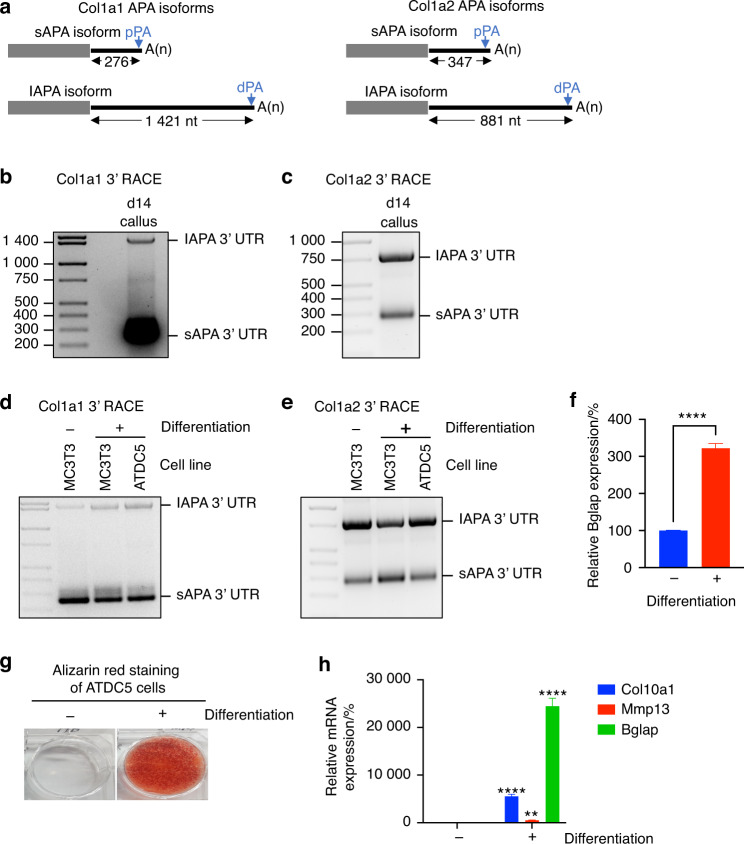
Long and short APA isoforms of the Col1a1 and Col1a2 mRNAs are expressed in the fracture callus as well as osteogenic and chondrogenic cell lines. **a** Schematic of the lAPA and sAPA isoforms of Col1a1 (left) and Col1a2 (right). The determined size of the 3′ UTR of each isoform is shown. **b** Representative gel image of Col1a1 3′ RACE-PCR products showing the 2 APA isoforms. 3′ RACE was performed using RNA purified from d14 calli (3′ RACE procedure is shown in Fig. S8a, b). The 3′ end of each APA isoform was mapped by excising and sequencing the corresponding PCR band (Fig. S9a). **c** As in **b**, except 3′ RACE-PCR was performed on Col1a2 mRNA. Sequencing of the PCR products confirmed the lAPA and sAPA isoforms that were identified by InPAS (Fig. S9b). **d**, **e** As in **b** and **c**, respectively, except that RNA was purified from the indicated cell lines. ATDC5 cells were differentiated under conditions that promoted hypertrophic differentiation and mineralization (Fig. S10). **f** RT‒qPCR analysis of the expression of the osteoblast and mineralization marker bone gamma carboxyglutamate protein (Bglap) in undifferentiated and differentiated MC3T3 cells. The mRNA expression level of Bglap was normalized to that of β-actin, and the normalized level in undifferentiated cells was defined as 100. **g** Alizarin red staining of ATDC5 cells before (−) and after (+) 3 weeks of differentiation. Positive staining of differentiated cells indicated chondrocyte mineralization (Fig. S10). **h** As in **f** except that RNA was purified from ATDC5 cells shown in **g**, and the specified transcript levels were quantified. The results demonstrate high expression of chondrocyte hypertrophy and mineralization markers in the differentiated ATDC5 cells. Gel images are representative of three independent replicates. The bar graphs present the average of three independent replicates ± SEM. **P < 0.01; ****P < 0.000 1 using unpaired Student’s *t* test

Type I Collagen, which is the main component of the organic bone matrix, is a heterotrimer comprising two α1 chains (encoded by Col1a1) and one α2 chain (encoded by Col1a2).^30^ According to our RNA-seq data analyses, Col1a1 and Col1a2 are the most abundant transcripts in the d14 callus, and both are expressed as 2 main APA isoforms (Fig. 3a and Tables S4, S5, S7). To confirm these results, we mapped the 3′ end of both the Col1a1 and Col1a2 mRNAs by performing rapid amplification of cDNA 3′ ends (3′ RACE) (Fig. S8a, b) using RNA purified from d14 calli. Indeed, we identified two APA isoforms of the Col1a1 and Col1a2 mRNAs (Fig. 3a–c) and mapped their 3′ ends to CPSs that were consistent with the bioinformatics predictions (Fig. S9a, b). Interestingly, we identified the same Col1a1 and Col1a2 APA isoforms when we performed 3′ RACE on RNA that was isolated from undifferentiated or differentiated murine MC-3T3-E1 osteoblastic cells (Fig. 3d–f).

Abundant levels of type I collagen are also secreted by mineralizing chondrocytes (Fig. 1d).^1,3,23^ To investigate whether the expression of Col1a1 and Col1a2 APA isoforms is specific to bone cells or whether these isoforms are also expressed in hypertrophic chondrocytes, we differentiated ATDC5 chondrogenic cells under conditions that induced chondrocyte hypertrophy and mineralization (Figs. 3g, h and S10a, b). We detected the same APA isoforms in hypertrophic ATDC5 cells (Fig. 3d, e). Notably, we sequenced the 3′ RACE-PCR products of Col1a1 and Col1a2 in all these experiments and confirmed that the 3′ end of each of the lAPA and sAPA isoforms is conserved among the studied cell lines and calli (Fig. S9a, b). Accordingly, APA-mediated 3′ UTR shortening in different mineralized tissues and cell lines removes ∼80% and 60% of the 3′ UTRs of the Col1a1 and Col1a2 mRNAs, respectively.

### 3′ UTR shortening reverses the inhibition of Col1a1 and Col1a2 expression

**Fig. 4:**
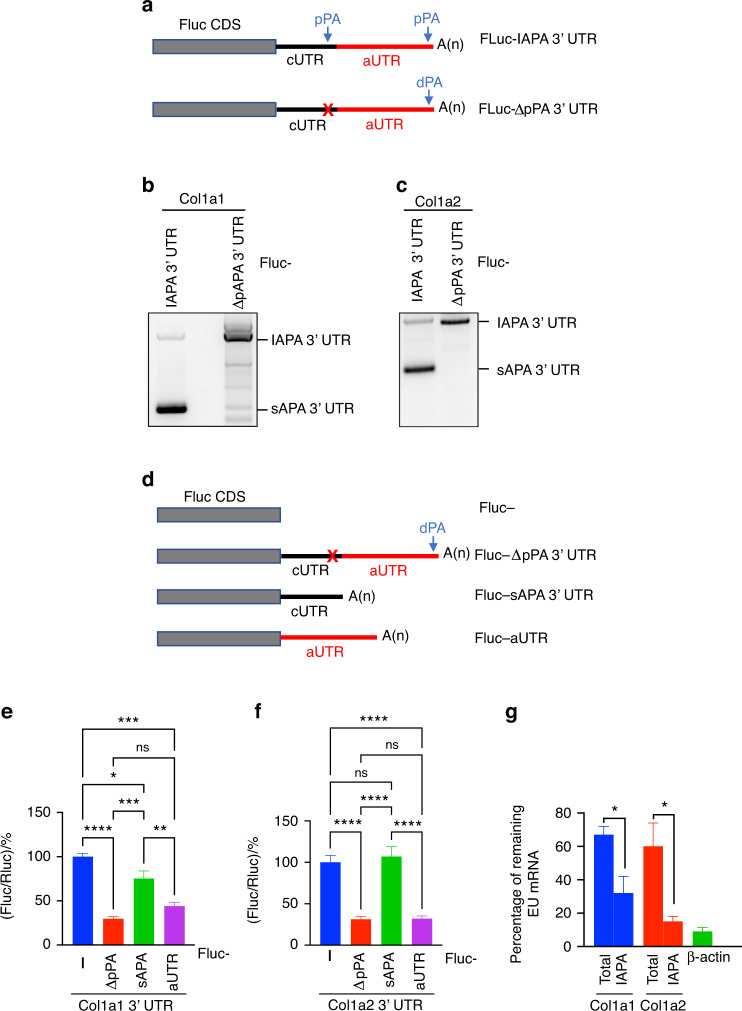
Shortening of the 3′ UTR promotes the expression of Col1a1 and Col1a2. **a** Schematic of FLuc reporters that contain either the 3′ UTR of the lAPA isoform (i.e., full-length 3′ UTR) of Col1a1 or Col1a2 (FLuc-lAPA 3′ UTR) or the deletion mutant in which the SSR was deleted (FLuc-ΔpPA 3′ UTR; X denotes the position of the deleted SSR). CDS: coding region. **b** 3′ RACE-PCR of FLuc mRNA was performed on RNA that was isolated from MC-3T3 cells transfected with Col1a1 3′ UTR reporters shown in **a**. PCR was performed using an FLuc-specific forward primer. The results indicate that deletion of the SSR in the FLuc-ΔpPA 3′ UTR reporter abrogated 3′ UTR shortening, resulting in the expression of FLuc mRNA as a single, long APA isoform. **c** As in **b**, except Col1a2 3′ UTR reporters were used. **d** Schematic of FLuc reporters that contain different isoforms or regions of the Col1a1 or Col1a2 3′ UTR. **e** Bar graph of the results of the dual luciferase assays that were performed on MC3T3 cells transfected with FLuc-Col1a1 3′ UTR reporters shown in **d**. FLuc activity was normalized to Renilla luciferase (RLuc) activity, and the FLuc/RLuc ratio in cells transfected with the “empty” FLuc (FLuc-) reporter was defined as 100. **f** As in **e** except that FLuc-Col1a2 3′ UTR reporters were used. *n* = 4–5 independent transfections, each measured in triplicate. **g** Bar graph of the results of the pulse-chase experiments used to measure RNA decay. MC3T3 cells were incubated with 5-ethynyl uridine (EU), which is an analog of uridine, for 2 h to label the nascent RNA during the “pulse step”. Cells were then either collected to determine the total level of EU-labeled mRNA (EU mRNA) or grown for 24 h in EU-free medium during the “chase step”. At each timepoint, EU RNA was purified (see Materials and methods), and the level of each of the specified EU mRNA was determined using RT‒qPCR. Primers that bind to the aUTR were used to specifically quantify the expression of the lAPA isoform of Col1a1 or Col1a2 (Table S14). The level of EU mRNA measured after the pulse step was defined as 100%, and the graph shows the % remaining after the 24-h chase step. The results demonstrate faster loss of the Col1a1 and Col1a2 lAPA isoforms compared to the total mRNA. β-actin mRNA was used as a positive control for EU mRNA decay, and consistent with its reported half-life (∼6.6 h),^50^ <10% remained after the 24-h chase. The gel images are representative of three independent replicates. The bar graphs present the average ± SEM. (ns) *P* > 0.05; *P < 0.05; **P < 0.01; ***P < 0.001; ****P < 0.000 1 using one-way analysis of variance (ANOVA) followed by Tukey’s post hoc test for **e** and **f**, and unpaired Student’s *t* test for **g**

To investigate the biological significance of the APA-mediated shortening of the Col1a1 and Col1a2 3′ UTRs, we performed mechanistic studies using MC-3T3 cells, which express APA isoforms of Col1a1 and Col1a2 that are identical to those expressed in the callus (Fig. 3b–e). We constructed a reporter containing the 3′ UTR of the Col1a1 lAPA isoform (lAPA 3′ UTR), which represents the full-length 3′ UTR, downstream of the firefly luciferase (FLuc) coding region (FLuc-lAPA 3′ UTR; Fig. 4a). As expected, when introduced into MC3T3 cells, this reporter expressed 2 APA isoforms of FLuc mRNA (Fig. 4b). The 3′ end of each of the 2 isoforms was identical to that of endogenous Col1a1 (Fig. S9a). Similarly, a reporter containing the lAPA 3′ UTR of Col1a2 mRNA expressed 2 APA isoforms of FLuc mRNA that were identical to those of endogenous Col1a2 (Figs. 4c and S9b). Accordingly, the FLuc-lAPA 3′ UTR reporters recapitulated the alternative cleavage and polyadenylation of the corresponding endogenous transcripts. We next examined the 3′ UTRs of both Col1a1 and Col1a2 mRNAs to define putative PAs that might modulate proximal cleavage and polyadenylation. In each transcript, we identified an A-rich simple sequence repeat (SSR) that contained overlapping canonical (AAUAAA) PAs that were located <50 nt upstream of the proximal CPS (Fig. S9a, b). Deletion of the SSR (FLuc-ΔpPA 3′ UTR reporter; Fig. 4a) almost completely abolished the 3′ UTR shortening of both the Col1a1 and Col1a2 reporters, resulting in the expression of each reporter as a single APA isoform with a full-length 3′ UTR (Fig. 4b, c). These results indicate that SSR-embedded pPAs are the main drivers of 3′ UTR shortening in both the Col1a1 and Col1a2 genes.

To investigate how 3′ UTR shortening impacts gene expression, we constructed an additional FLuc reporter containing the sAPA 3′ UTR of Col1a1 or Col1a2 (FLuc-sAPA 3′ UTR; Fig. 4d). We compared the reporter activities of the FLuc-ΔpPA 3′ UTR and FLuc-sAPA 3′ UTR constructs, and the results indicated strong inhibition of FLuc activity (>70%) by the lAPA 3′ UTR of either Col1a1 or Col1a2 (Fig. 4e, f), while the repression that was caused by the sAPA 3′ UTR of Col1a1 and Col1a2 was very moderate (∼25%) (Fig. 4e) and not significant (Fig. 4f), respectively. These results demonstrate that the aUTR region, which is removed during 3′ UTR shortening, exerts a potent inhibitory effect on the expression of Col1a1 and Col1a2. Consistent with this observation, a reporter that contains the aUTR region of Col1a1 or Col1a2 (FLuc-aUTR; Fig. 4d) repressed Fluc activity to the same extent as the full-length 3′ UTR (Fig. 4e, f). To corroborate the reporter results and investigate the impact of 3′ UTR shortening on endogenous transcripts, we performed pulse-chase experiments and assessed the stability of endogenous Col1a1 and Col1a2 APA isoforms. The results indicated substantially faster degradation of the lAPA isoforms than the sAPA isoforms (Fig. 4g). Thus, 3′ UTR shortening enhances Col1a1 and Col1a2 expression via, at least in part, the stabilization of the transcripts.

### Shortening of the 3′ UTR enhances Col1a1 and Col1a2 expression by removing miR-29a-3p binding sites

**Fig. 5:**
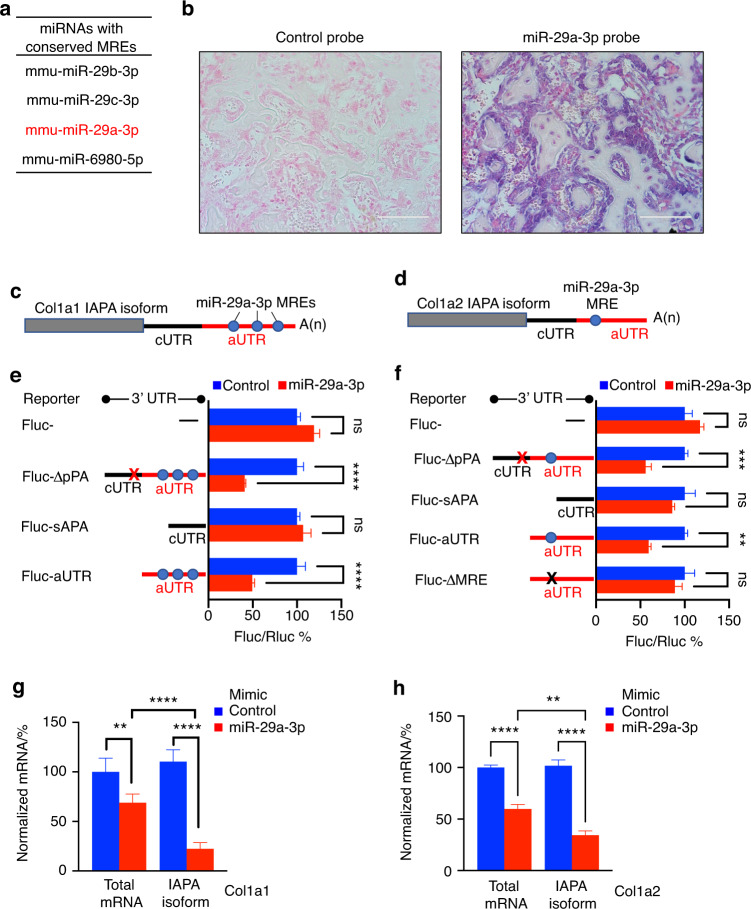
miR-29a-3p mediates the degradation of the lAPA, but not sAPA, isoforms of Col1a1 and Col1a2 mRNAs. **a** List of miRNAs that have conserved MREs in the 3′ UTR of mouse Col1a1 mRNA (identified by both TargetScan and miRDB) (Tables S8 and S9). miR-29a-3p (highlighted in red) exhibited the highest expression among the 4 miRNAs in the d14 callus (Table S10). **b** ISH using either a control (left) or miR-29a-3p (right) probe. The results show intense staining (blue) of miR-29a-3p in the woven bone area of the d14 callus. **c** Schematic of the lAPA isoform of Col1a1 mRNA showing the putative miR-29a-3p MREs in the aUTR. **d** As in **c** except Col1a2 mRNA is shown. **e** Bar graph of the results of dual luciferase assays that were performed in MC3T3 cells transfected with the indicated Col1a1 FLuc reporters along with either a control (blue) or miR-29a-3p (red) mimic. FLuc activity was normalized to RLuc activity, and the FLuc/RLuc ratio in cells transfected with the control mimic was defined as 100. *n* = 4–5 independent transfections, each measured in triplicate. **f** As in **e** except the cells were transfected with Col1a2 FLuc reporters. In the FLuc-ΔMRE reporter, we replaced the binding region of the miR-29a-3p seed sequence with a random sequence (denoted by “X”). **g** RT‒qPCR quantification of the expression of either total mRNA or the lAPA isoform of Col1a1 in MC3T3 cells transfected with control (blue) or miR-29a-3p (red) mimic. Primers that bind within the aUTR region were used to specifically amplify and quantify the levels of lAPA isoform (Table S14). The expression level was normalized to that of β-actin mRNA, and the normalized level in control mimic-transfected cells was defined as 100. *n* = 5. **h** As in **g** except that the expression levels of total mRNA and the lAPA isoform of Col1a2 were quantified. The bar graphs present the average ± SEM. (ns) *P* > 0.05; **P < 0.01; ***P < 0.001; ****P < 0.000 1. The significance of the difference was calculated using unpaired Student’s *t* test in **e** and **f** and two-way ANOVA in **g** and **h**

To define the inhibitory elements that are embedded within the aUTR of Col1a1 mRNA, we searched for putative miRNA response elements (MREs) among other factors. Among the top scoring miRNAs with conserved MREs in Col1a1 mRNA, four were identified by both TargetScan^31^ and miRDB;^32^ these four miRNAs included three members of the miR-29 family and miR-6980-5p (Fig. 5a and Tables S8 and S9). In fact, the miR-29 family members have been reported to inhibit the expression of Col1a1 with variable efficiency in different tissues.^33–36^. miRNA sequencing (miR-seq) of small RNAs that were isolated from d14 calli identified miR-29a-3p as one of the 15 most highly expressed miRNAs, generating ∼20 000 counts per million (CPM) (Table S10). The expression level of miR-29a-3p was ∼8-fold and 34-fold higher than that of miR-29c-3p and miR-29b-3p, respectively (Table S10). On the other hand, miR-6980-5p was expressed at very low levels, generating <10 CPM (Table S10). Thus, we focused our subsequent studies on miR-29a-3p. In situ hybridization confirmed the high expression of miR-29a-3p in woven bone osteoblasts and lining cells (Fig. 5b). Interestingly, miR-29a-3p has three MREs in the 3′ UTR of Col1a1 mRNA, all of which are in the aUTR (Figs. 5c and S11a and Tables S8 and S9). miR-29a-3p also has a single MRE in the aUTR of Col1a2 mRNA (Figs. 5d and S11b and Tables S8 and S9), suggesting that miR-29a-3p suppresses the expression of both Col1a1 and Col1a2 via MREs embedded in the aUTR. To further test this hypothesis, we used the same FLuc reporters that we constructed to express different isoforms and to contain different regions of the Col1a1 or Col1a2 3′ UTR (Fig. 5e, f). A miR-29a-3p mimic effectively repressed the reporters that contained the ΔpPA 3′ UTR or aUTR of either Col1a1 or Col1a2 by ≥50% (Fig. 5e, f). In contrast, the miR-29a-3p mimic had no effect on the sAPA reporters (Fig. 5e, f). Mutating the single MRE in the Col1a2 aUTR (FLuc-ΔMRE) abolished the inhibitory effect of miR-29a-3p (Fig. 5f). These data confirm that miR-29a-3p effectively inhibits the expression of Col1a1 and Col1a2 by binding to the aUTR. To corroborate the FLuc reporter data, we examined the effect of the miR-29a-3p mimic on endogenous transcripts. The mimic downregulated the total level of endogenous Col1a1 mRNA by ∼30% but exerted substantially stronger inhibitory effects on the lAPA isoform, decreasing its expression by ∼75% (Fig. 5g). Comparable results were obtained with endogenous Col1a2 mRNA (Fig. 5h). Consistent with this, although a miR-29a-3p inhibitor did not significantly change the total level of endogenous Col1a1 or Col1a2 mRNA, it increased the expression level of the lAPA isoforms of Col1a1 and Col1a2 by ∼1.8- and 2.3-fold, respectively (Fig. S12a, b). Taken together, these results highlight the role of 3′ UTR shortening in reversing the inhibition of Col1a1 and Col1a2 expression in biological contexts where miR-29a-3p, and possibly other members of the miR-29 family, are highly expressed.

### Endochondral ossification is accompanied by prevalent 3′ UTR shortening

**Fig. 6:**
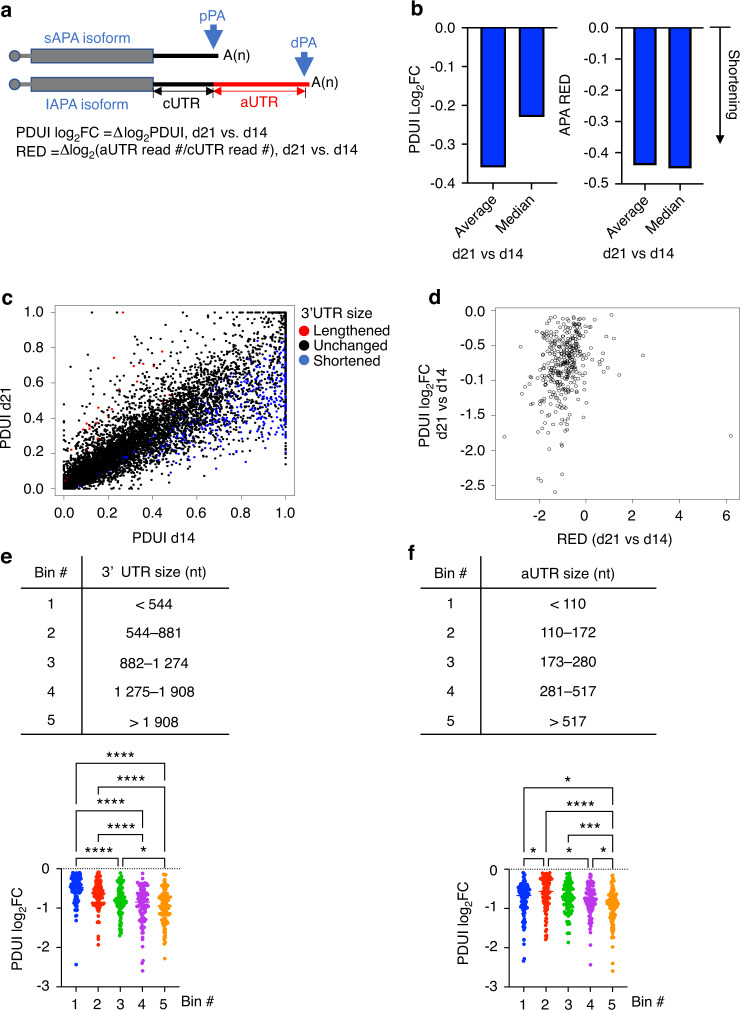
Prevalent 3′ UTR shortening accompanies endochondral ossification. **a** General schematic presentation of APA isoforms showing representative proximal and distal PAs (pPA and dPA, respectively). PDUI Log_2_FC and RED values were calculated by InPAS and APAlyzer, respectively, as indicated. **b** Average and median PDUI log_2_FC (left) and RED (right) values were calculated by InPAS and APAlyzer, respectively, for all the analyzed genes (Tables S4 and S5). The negative average and median values indicate a global trend of 3′ UTR shortening on d21 relative to d14. **c** A scatter plot showing changes in 3′ UTR length on d21 compared to d14. Each dot represents a gene, and significantly lengthened or shortened 3′ UTRs are shown in red and blue, respectively. Significance was defined as a *P*_adj_ < 0.05 and >20% change in the expression ratio of the 2 APA isoforms. **d** Correlation between InPAS-generated PDUI log_2_FC and APAlyzer-generated RED values for genes that exhibited significant 3′ UTR shortening on d21 relative to d14 (shown in blue in “**c**”). **e** (Top): Genes that exhibited significant 3′ UTR shortening were categorized into 5 similarly sized bins based on their 3′ UTR length. (bottom): scatter plot showing the relationship between 3′ UTR length and the extent of 3′ UTR shortening (presented as PDUI log_2_FC). Each dot represents a gene. The data are presented as the average ± SEM. **f** As in **e** except that the genes were categorized based on their aUTR length. *P < 0.05; ***P < 0.001, ****P < 0.000 1 using one-way ANOVA followed by Tukey’s post hoc test

Our RNA-seq data indicate that the progression of endochondral ossification is associated with increased cell proliferation (Figs. 1i and S5 and S6 and Tables S2 and S3). As rapidly proliferating cells exhibit widespread 3′ UTR shortening,^8,10^ we hypothesized that 3′ UTR shortening is prevalent during endochondral ossification. To investigate this, we used the InPAS to quantify changes in the ratios of APA isoform expression on d21 compared to d14 (InPAS calculates changes as log_2_fold-change in PDUI values; Fig. 6a and Table S4), and we corroborated the results using the APAlyzer (calculates changes as relative expression difference (RED) values; Fig. 6a and Table S5). Positive PDUI log_2_FC or RED values indicate higher ratios of lAPA isoform expression (i.e., lengthening events), while negative values indicate shortening events. Both InPAS and APAlyzer identified a global trend of 3′ UTR shortening on d21 relative to d14 (Fig. 6b). When we limited the InPAS analysis to transcripts that exhibited significant changes in 3′ UTR length (*P*_adj_ < 0.05 and >20% change in the expression ratio of the 2 APA isoform), the results demonstrated that the number of significant shortening events was ∼24-fold higher than the number of significant lengthening events (Fig. 6c); these results provided further evidence of substantial 3′ UTR shortening. APAlyzer confirmed these results (Fig. S13a, b) and corroborated ∼95% of the significant shortening events that were identified by InPAS (Fig. 6d). We also identified APA events that occur in introns (intronic polyadenylation; IPAs), but we did not observe any trend of IPA activation or suppression on d21 relative to d14 (Fig. S13c, d and Table S11). Importantly, GO analysis^37^ of the genes that exhibited significant 3′ UTR shortening demonstrated enrichment in pathways associated with cell cycle and proliferation, cellular immune response, cellular growth, assembly, organization, cell-to-cell signaling, and transcriptional regulation (Tables S12 and S13).

To investigate whether the length of the 3′ UTR correlates with the extent/frequency of pPA utilization, we classified transcripts that underwent significant 3′ UTR shortening into 5 categories based on the lengths of their 3′ UTRs (Fig. 6e, top) or aUTRs (Fig. 6f, top), and we calculated the average PDUI log_2_FC for each category. We found that genes displayed a greater degree of pPA utilization (i.e., higher frequency of 3′ UTR shortening) as the length of the 3′ UTR (Fig. 6e, bottom) or aUTR (Fig. 6f, bottom) increased.

### Transcripts that undergo 3′ UTR shortening are expressed at higher levels

**Fig. 7:**
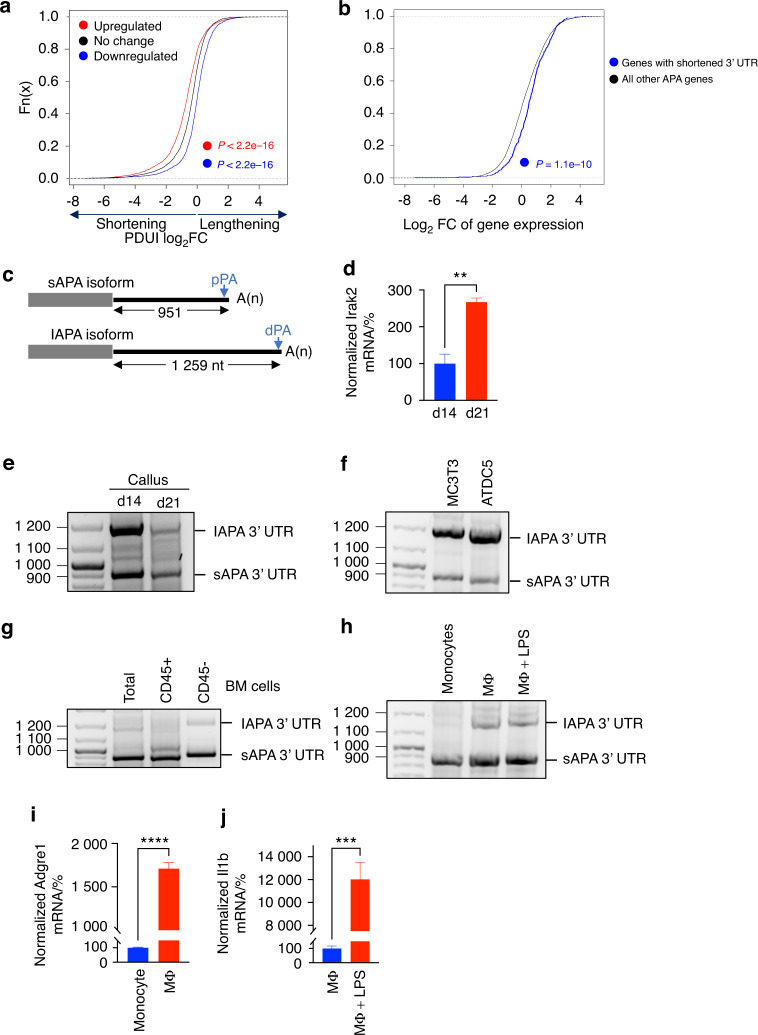
3′ UTR shortening is associated with increased gene expression. **a** Cumulative distribution function (CDF) curves of the PDUI log_2_FC values (generated by InPAS) of genes whose expression was significantly upregulated (red line), significantly downregulated (blue line), or unchanged (black line) on d21 relative to d14. A significant change in gene expression was defined as *P*_adj_ < 0.05. All the InPAS-identified APA events are included in the analysis (see Fig. S14a for APA events identified by APAlyzer). *p* values (K-S test) indicating the significance of the difference between the red or blue and black genes are indicated. **b** CDF curves comparing the expression of genes that exhibited significant 3′ UTR shortening according to InPAS (blue line) and all other genes (black line). *P* values (K-S test) are based on comparisons of the blue and black genes (see Fig. S14b for APAlyzer data). **c** Schematic of the lAPA and sAPA isoforms of Irak2. **d** RT‒qPCR analysis of Irak2 expression in d14 and d21 calli. The mRNA expression level of Irak2 was normalized to that of β-actin, and the normalized level in d14 calli was defined as 100. Representative gel image of Irak2 3′ RACE-PCR products from d14 and d21 calli (**e**), differentiated MC3T3 and ATDC5 cells (**f**), total, CD45^+^, and CD45^−^ bone marrow (BM) cells (**g**), monocytes, macrophages (MΦ), and LPS-stimulated MΦ (**h**). **i** As in **d**, except the expression of the MΦ marker Adgre1 was measured. The results indicate successful in vitro differentiation of monocytes to MΦ. **j** As in **d** except that Il1b mRNA expression was measured. The results indicate upregulated expression of the proinflammatory Il1b gene in response to LPS stimulation. The bar graphs present the average ± SEM. **P < 0.01; ***P < 0.001; ****P < 0.000 1 using unpaired Student’s *t* test

The 3′ UTR plays crucial roles in regulating the steady-state level of a transcript. We investigated the global impact of d14-to-d21 3′ UTR shortening on gene expression and found that genes whose expression was upregulated on d21 relative to d14 displayed a greater degree of 3′ UTR shortening than genes that showed no change in expression (Figs. 7a and S14a). In a striking contrast, downregulated genes showed the opposite trend and exhibited 3′ UTR lengthening (Figs. 7a and S14a). Consistent with this global trend, when we limited our analysis to genes that underwent significant 3′ UTR shortening (*P*_adj_ < 0.05 and >20% change in the APA isoform expression ratio; Tables S4 and S5), we found their expression to be significantly higher than that of all other genes (Figs. 7b and S14b). These results highlight the impact of 3′ UTR shortening on enhancing gene expression during endochondral ossification.

Interleukin-1 receptor-associated kinase 2 (Irak2), a serine/threonine kinase that associates with the interleukin-1 receptor upon activation, is one of the genes that was identified by InPAS and APAlyzer to undergo significant 3′ UTR shortening during endochondral ossification (Fig. 7c and Tables S4 and S5). Shortening of the Irak2 3′UTR was accompanied by a significant increase in gene expression, as demonstrated by RNA-seq data (Table S1) and RT‒qPCR analysis (Fig. 7d), and this result was consistent with the global trend. 3′ RACE experiments confirmed the expression of 2 main APA isoforms of Irak2 in the callus (Fig. 7e), mapped the 3′ end of each isoform (Fig. S14c), and confirmed the shortening of the Irak2 3′ UTR on d21 relative to d14 (Fig. 7e). We then investigated whether the ratio of Irak2 APA isoform expression varies among cell types given that the callus is made up of heterogeneous cell populations that vary between d14 and d21 (Fig. 1). The enrichment of bone marrow and immune cells is greater on d21 than d14 (Figs. 1i and S5 and S6 and Tables S2 and S3), so to determine whether any APA isoform of Irak2 is specifically upregulated in bone marrow cells, we compared the APA isoforms that were expressed in the total bone marrow (flushed from the callus on d21) to those that were expressed in osteogenic and chondrogenic cell lines. We found that while the lAPA isoform is the major isoform that is expressed in differentiated MC3T3 and ATDC5 cells, the sAPA isoform is the major isoform that is expressed in total bone marrow cells, which exhibited barely detectable expression of the lAPA isoform (Fig. 7f, g). To further identify the APA isoforms expressed in different bone marrow cell populations, we fractionated the bone marrow, using fluorescence-activated cell sorting (FACS), into immune (CD45^+^) and nonimmune (CD45^−^) cells; we found that the sAPA isoform is the major isoform in both populations (Fig. 7g). Then, to investigate Irak2 APA isoform expression in bone marrow myeloid cells, we isolated primary monocytes from the bone marrow and differentiated them into macrophages in vitro. We found that monocytes almost exclusively expressed the sAPA isoform (Fig. 7h), while in vitro differentiation into macrophages resulted in mild 3′ UTR lengthening (Fig. 7h, i). Activation of mature macrophages using lipopolysaccharide (LPS) did not change the ratio of APA isoform expression (Fig. 7h, J). Accordingly, bone marrow cells, which populate the woven bone area and the re-established intramedullary cavity on d21, are a major source of the Irak2 sAPA isoform; these results explain, at least in part, the observed d14-to-d21 shortening of the Irak2 3′ UTR.

## Discussion

Secondary bone healing via endochondral ossification is the most common healing mechanism observed in the clinic.^1–3,23^ During secondary healing, stromal progenitors differentiate into chondrocytes due to the hypoxic conditions that develop in the fracture gap as a result of blood vessel rupture.^1–3^ Chondrocytes proliferate to form fibrocartilaginous calli and then undergo hypertrophic differentiation. Hypertrophic chondrocytes secrete extracellular proteins, including Col I and Col X, which facilitate the mineralization of the soft callus, and metalloproteinases, including MMP13, which reorganize the extracellular matrix.^1–3,23^ Mineralization of the soft callus enables the formation of new blood vessels across the fracture site, and hypertrophic chondrocytes are gradually replaced by newly formed bone via the process of endochondral ossification.^1–3^ This process resembles the endochondral formation of long bone from hypertrophic chondrocytes during development.^38^ Our data provide a comprehensive comparison between the transcriptome of the callus on d14, which represents the terminal stages of soft-callus hypertrophy and mineralization, and that of the callus on d21, the time point at which the fracture gap is completely bridged by woven bone (Figs. 1 and S1–6 and Tables S1–S3). The genes that were expressed in the callus on d14 were enriched in pathways involved in cartilage and connective tissue development, ossification of the fibrocartilaginous callus, and extracellular matrix reorganization (Figs. 1i and S5 and S6 and Tables S2 and S3). Replacement of mineralizing hypertrophic chondrocytes by developing woven bone was accompanied by a significant increase in the immune and inflammatory responses, including leukocyte activation, adaptive immune response, cytokine production, cell cycle, nuclear division, and DNA replication (Figs. 1i and S5 and S6 and Tables S2 and S3). Accordingly, as endochondral ossification proceeds, the callus becomes populated by rapidly dividing cells. It is noteworthy that the intramedullary cavity is re-established and invaded by bone marrow on d21, which may partially account for the observed increase in the immune response. However, some of the transcriptomic changes that were identified, particularly those related to the adaptive immune system, may also suggest the active infiltration of immune cells into the callus on d21. The roles of different immune cell populations during these late phases of healing have yet to be identified.

The boundaries of the 3′UTRs of most RNA polymerase-derived transcripts are generated cotranscriptionally by the recognition of PAs and endonucleolytic cleavage at CPSs, followed by the addition of poly (A) tails.^8^ Most mammalian genes have multiple PAs that are tandemly located in the last exon, so mRNAs are expressed as APA isoforms that have the same CDS but distinct 3′UTRs. Notably, during evolution, the 3′ UTR size has increased from ∼140 nts in *Caenorhabditis elegans* to 1–2 kilobases in humans,^10^ resulting in a higher number of 3′ UTR-embedded elements that regulate mRNA processing and metabolism.^7,8,10^ Thus, APA has significant functional consequences and contributes to the diversification of the transcriptome and the establishment of cell identity.^39^ Global reprogramming of APA is a characteristic of several types of cancer, and specific APA events are implicated in malignancies and autoimmune diseases.^8,15,40^ Despite the increased interest in APA, its roles in bone homeostasis and repair remain unknown. Our data highlighted the dynamic nature of the 3′ UTR landscape in the callus across different healing phases and identified thousands of genes that are expressed as APA isoforms in the healing callus (Fig. 2 and Tables S4 and S5). The rapidly proliferating cells that gradually populated the callus during endochondral ossification showed a global trend of 3′ UTR shortening (Figs. 6b, c and S13a, b), which is consistent with the established association of cell proliferation with APA control.^8,10^ Shortening of the 3′ UTR clearly correlated with the increased expression of hundreds of genes that play critical roles in bone repair (Figs. 7a, b and S14a, b and Tables S12 and S13); these findings emphasized the role of APA in the posttranscriptional regulation of gene expression during endochondral ossification. Crucially, APA can also occur in introns (IPA). Although IPA has been reported to be prevalent in immune cells,^41^ we did not observe a trend of IPA activation on d21 relative to d14 (Fig. S13c, d), despite an obvious increase in immune cell recruitment and activity over this period. However, we identified several IPA events at each timepoint (Table S11), and these events are expected to stimulate mRNA degradation via nonsense-mediated decay or result in the expression of truncated proteins. The biological significance of these IPA events in fracture healing remains uncharacterized and will be interesting to address in future studies.

Regulation of APA occurs in a stimulus- and cell/tissue-specific manner. An example of the latter is Irak2, which undergoes significant 3′ UTR shortening as healing proceeds from d14 to d21 (Fig. 7e and Tables S4 and S5). sAPA is the major Irak2 APA isoform expressed in bone marrow (Fig. 7g), while lAPA is the major isoform expressed in differentiated MC3T3 and ATDC5 cells (Fig. 7f). The major Irak2 APA isoform switches from lAPA on d14 to sAPA on d21 as the bone marrow population is re-established on d21 (Fig. 7e). In myeloid cells, sAPA is the sole isoform expressed in monocytes and the major isoform expressed in macrophages and activated macrophages (Fig. 7h). Given that there is a clear correlation between Irak2 mRNA 3′ UTR shortening and increased Irak2 mRNA steady-state abundance (Fig. 7d, e and Tables S1, S4, S5), 3′ UTR shortening might be one of the factors that posttranscriptionally enhances Irak2 expression in monocytes and macrophages, and in turn, Irak2 directs the expression of inflammatory cytokines.^42^

The growing interest in APA as an important mechanism for transcriptome diversification has spurred advances in technologies to quantify APA isoforms. Various experimental approaches have been developed that have enabled the deep sequencing and annotation of Poly(A) sites in human and mouse genomes, leading to the establishment of several PolyAsite databases that integrate results from different 3′ end sequencing approaches.^8^ However, the volume of data that has been generated thus far using 3′ end sequencing and the scope of cell/tissue types covered by these data are very limited compared with generic RNA-seq data. Therefore, subsequent bioinformatics efforts have been made to identify and quantify PA usage based on RNA-seq data. Algorithms have been developed to identify PAs as positions that exhibit a clear decrease in RNA-seq reads.^15^ Different RNA-seq sets have been analyzed using these algorithms, including analysis of The Cancer Genome Atlas using DaPars, which led to the identification of global 3′ UTR shortening in cancer cells.^15,16^ These different algorithms perform the same task using different approaches, but direct comparison of their relative accuracy in identifying and quantifying APA isoforms is lacking. One limitation of these algorithms is their reliance on identifying variations in the RNA-seq coverage profile around tandem PAs, which might be caused by factors other than APA. Furthermore, RNA-seq read coverage is inherently nonuniform along transcripts, which increases the rate of false positives in the identification of de novo PAs. These limitations can be addressed by focusing the analysis of PAs that have already been annotated, an approach that has been applied by different APA analysis tools, including InPAS^17,18^ and APAlyzer,^19^ which we utilized in this work. InPAS and APAlyzer identified the same pattern of changes in 3′ UTR length during endochondral ossification, and the major findings obtained with these tools were consistent. In general, APAlyzer identified more APA events than InPAS did (Figs. 6c and S13b and Tables S4 and S5), but we focused our studies on the APA events that were detected by both tools as a means to increase robustness. It is also noteworthy that limiting APA analysis to already annotated PAs limits the coverage of APA events and restricts the identification of de novo PAs. In conclusion, we used different filters and approaches to increase the accuracy of PA identification, even though this might have resulted in underestimating the number of APA events in the callus.

PA sequences have likely been selected during evolution to establish specific ratios of APA isoform expression in each cell/tissue. Consistent with this, during cell differentiation, genes with multiple APA isoforms undergo changes in isoform expression ratios to achieve patterns that maintain the required tissue-specific expression levels.^39^ Our data indicate that the sAPA/lAPA ratio of Col1a1 or Col1a2 transcript in mineralizing osteoblasts was comparable to that in mineralizing chondrocytes (Fig. 3d, e), indicating the importance of establishing a particular sAPA/lAPA ratio to maintain the required protein expression level of Col I in mineralizing cells. Shortening of the 3′ UTR in Col1a1 and Col1a2 was primarily mediated by one or more of the SSR-embedded (AAUAAA) hexamers that follow the general roles of canonical PAs: they are embedded in an A/U-rich region that is located ≥21 nucleotides upstream of the identified proximal CPSs (Figs. 4a–c and S9a, b). Luciferase assays and analyses of the decay rate of endogenous transcripts indicate that 3′ UTR shortening substantially enhanced the stability of Col1a1 and Col1a2 mRNAs and induced gene expression via, at least in part, the removal of miR-29a-3p MREs that are embedded in the aUTR (Figs. 4d–g and 5c–h). These similar mechanisms that regulate both Col1a1 and Col1a2 mRNA expression suggest an evolutionary path that culminated in parallel regulatory elements being embedded in the transcripts of two different genes that encode subunits of the same protein.

Members of the miR-29 family (miR-29), which have overlapping targets, stimulate the differentiation of MC3T3 cells via the direct inhibition of the expression of the osteogenesis inhibitory factors HDAC4, TGFβ3, ACVR2A, CTNNBIP1, and DUSP2.^35^ Consistent with the osteogenic roles of miR-29, our data indicate high expression of miR-29a-3p in newly formed osteoblasts and bone lining cells (Fig. 5b). Furthermore, the expression of miR-29b has been reported to increase during MC3T3 differentiation.^35^ miR-29 also inhibits the expression of Col1a1 to a variable extent in different tissue/cell types.^33,34,36,43^ However, the repressive effect of miR-29 on Col1a1 expression is mild or undetectable in MC3T3 cells, especially during early phases of differentiation.^35^ This raised a long-standing question of how Col1a1 evades the repressive effects of miR-29 during osteogenic differentiation.^35^ In fact, it is counterintuitive that miR-29 promotes osteogenesis and, at the same time, inhibits the expression of Col I, which is an essential protein for differentiation and mineralization. Our data provide a likely answer to this long-standing question and propose APA-mediated 3′ UTR shortening as the mechanism that enables the Col1a1 and Col1a2 genes to be expressed at high levels in the presence of high concentrations of pro-osteogenic miR-29a-3p. Importantly, the pathological overexpression of Col I induces fibrosis, and a miR-29 mimic can act as an antifibrotic agent in different tissues by repressing the expression of Col I and other fibrotic proteins.^33,34,36,43^ More than one miR-29 mimic is now in clinical trials as investigative next-generation treatments for idiopathic pulmonary fibrosis.^44^ However, the anti-fibrotic effects of miR-29 and its impact on Col I expression vary substantially among tissues,^33,34,36,43^ suggesting that tissue-specific APA-mediated shortening of the Col1a1 and Col1a2 3′UTRs might contribute to inhibiting the anti-fibrotic effects of miR-29 in certain tissues.

In summary, our data reveal a novel role of APA as a posttranscriptional regulatory mechanism that participates in shaping the transcriptome and regulating gene expression during bone regeneration.

## Materials and methods

### Animals

All the animal protocols were approved by the University Committee on Animal Resources (IACUC) at the Pennsylvania State University College of Medicine. Adult male C57BL/6J mice at 14 weeks of age were purchased from The Jackson Laboratory (Bar Harbor, ME, USA) and allowed to acclimate for 2 weeks. The mice were housed in ventilated cages and provided ad libitum access to pelleted food and water. The mouse facility was maintained on a 12-h light/dark cycle, at a temperature of 21.1 °C to 22.8 °C, and 30% to 70% humidity.

### Mid-diaphysis tibial fracture surgery and tissue harvest

Mid-diaphyseal tibial fractures were established in the right hindlimb and stabilized by an intramedullary nail as previously described.^23,24^ X-ray images were collected postoperatively and at harvest time to confirm proper alignment at the fracture site. For samples that were subjected to safranin-O/fast green staining, IF staining, or ISH, the fractured hindlimb was harvested from the mid-femur to the tibiotalar joint to avoid disturbing the callus tissue, and the surrounding muscle tissues were removed, leaving only the soft tissue that was in direct contact with the bone to support the callus during the subsequent processing steps. The isolated bone was then fixed in 10% (v/v) neutral buffered formalin (Thermo Fisher Scientific), decalcified in 14% w/v EDTA tetrasodium (Thermo Fisher Scientific), and embedded in paraffin. The harvest time was determined based on published studies about the time course of healing in this model.^23,24^

### Safranin-O/fast green and IF staining

Sagittal sections with thicknesses of 5 µm that spanned the center of the fracture callus were obtained and stained with hematoxylin/Safranin-O/Fast Green as previously described.^23^ Imaging was performed using the OsteoMeasure system (OsteoMetrics Inc.). For IF staining, Col I and Col II were stained according to a 2-step staining protocol, while MMP13 and Col X were stained according to a 3-step protocol as we previously described.^23^ Mounting and nuclear staining were performed using ProLongTM Gold antifade reagent with DAPI (Invitrogen). Images were captured using a Zeiss Axio Observer 7 upright wide-field microscope (Carl Zeiss Microscopy GmbH).

### RNA purification, reverse transcriptase (RT)-qPCR, and ISH

The soft tissues that surrounded the callus were completely removed, the callus tissue was isolated (avoiding the surrounding cortical bone) and snap frozen in liquid nitrogen, and RNA was purified as we previously described.^45^ RNA was purified from cell lines, primary monocytes, and macrophages using an RNeasy Kit (Qiagen). cDNA was prepared from <1 μg RNA using SuperScript IV Reverse Transcriptase (Thermo Fisher Scientific) and qPCR-amplified using TaqMan Fast Advanced Master Mix and TaqMan Gene-Expression Assays (Thermo Fisher Scientific). For lAPA isoforms, qPCR was performed using PowerUp SYBR Green Master Mix (Thermo Fisher Scientific), and the primers are listed in Table S14. ISH was performed using a miRCURY LNA miRNA ISH kit (Qiagen) according to the manufacturer’s instructions.

### Library preparation and RNA sequencing

RNA-seq was performed on three biological replicates (i.e., callus tissues) for each timepoint. cDNA libraries were prepared using the Illumina® Stranded mRNA Prep and Ligation kit (Illumina) according to the manufacturer’s instructions. Briefly, poly(A) RNA was purified from 200 ng total RNA using oligo (dT) beads. The purified fraction was then subjected to fragmentation, reverse transcription, end repair, 3′ adenylation, and adapter ligation, followed by PCR amplification and SPRI bead purification (Beckman Coulter). The unique dual index sequences (IDT® for Illumina® RNA UD Indexes Set A, Ligation, Illumina) were incorporated into the adapters for multiplexed high-throughput sequencing. The size distribution and concentration of the final product were analyzed using a BioAnalyzer High Sensitivity DNA Kit (Agilent Technologies). The libraries were pooled, diluted to 3 nmol·L^−1^ using 10 mmol·L^−1^ Tris-HCl, pH 8.5, and denatured using the Illumina protocol. The denatured libraries were loaded onto an S1 flow cell (Illumina NovaSeq 6000) and run for 2 × 53 cycles according to the manufacturer’s instructions.

### Analysis of RNA-seq data and APA

Multimultiplexed and adapter-trimmed sequencing reads were generated using Illumina bcl2fastq (released version 2.20.0.422), and no mismatches were allowed in the index read. BBDuk (ourceforge.net/projects/bbmap/) was used to trim/filter low-quality sequences using the “qtrim = lr trimq = 10 maq = 10” option. The filtered reads were aligned to the mouse reference genome (GRCm38) using HISAT2 (version 2.1.0)^46^ applying --no-mixed and --no-discordant options. Read counts were calculated using HTSeq^47^ by supplementing Ensembl gene annotation (GRCm38.78). EdgeR^48^ was used to fit the read counts to the negative binomial model along with a generalized linear model (GLM), and differentially expressed genes were identified using DESeq2.^26^ GO and functional analyses were performed by Ingenuity Pathway Analysis,^37^ DAVID,^29^ iDEP,^28^ and/or GAGE.^27^ Heatmaps and volcano plots were prepared using iDEP.^28^

APA was analyzed using two different software programs: InPAS v3.14 and APAlyzer v4.1. Default settings were used for each program. For InPAS, the PDUI for each gene was calculated as the ratio of lAPA RPM to the total RPM of the two main APA isoforms, and PDUI log2FC was calculated as the difference in log_2_PDUI between d21 and d14. APA events were considered significant when they had an adjusted *P* value <0.05 and >20% change in the expression ratio of the two main APA isoforms. For APAlyzer, the RED value was calculated as the difference in log2 (RPM ratio) of the two APA isoforms (i.e., log2(aUTR read number/cUTR read number)) between d21 and d14. The aUTR length was calculated as the difference between the two defined CPSs in the 3ʹUTR. To compare the gene expression of different APA gene sets (Figs. 7a, b and S14a, b), genes were classified into significantly upregulated, no change, or significantly downregulated gene sets based on counting the reads that mapped to CDS alone. The differential expression of CDS reads was analyzed using the APAlyzer package. This approach was used to avoid confounding the data by including reads from the 3′ UTR that is different among APA isoforms. However, using total RNA reads (generated by DESeq2) instead of CDS reads generated correlations comparable to those shown in Figs. 7a, b and S14a, b (data not shown). CDF curves were created using the R package ggplot2.

### Library preparation and miRNA sequencing

Small RNA-sequencing libraries were prepared from 250 ng of total RNA using the QIAseq miRNA Library Kit (QIAGEN) according to the manufacturer’s instructions. This system offers a built-in Unique Molecular Identifier (UMI) application that eliminates the possibility of PCR duplicates in being present in sequencing datasets and, therefore, facilitates unbiased gene expression profiling. Unique barcode sequences were incorporated into the adapters for multiplexed high-throughput sequencing. The size distribution and concentration of the final product were assessed using a BioAnalyzer High Sensitivity DNA Kit (Agilent Technologies). Pooled libraries were diluted to 2 nM in EB buffer (Qiagen), denatured using the Illumina protocol, diluted to 10 pmol·L^−1^ using prechilled hybridization buffer, loaded onto a TruSeq v2 Rapid flow cell (Illumina HiSeq 2500), and run for 75 cycles using a single-read recipe according to the manufacturer’s instructions. Demultiplexed sequencing reads were generated using Illumina bcl2fastq (released version 2.20.0.422, Illumina), and no mismatches were allowed in the index read. Primary read mapping and UMI analysis were conducted using the GeneGlobe Data Analysis Center (QIAGEN).

### Cell culture and transfection

MC3T3-E1 cells were obtained from ATCC, cultured in α-modified Eagle’s medium (α-MEM) supplemented with 10% fetal bovine serum (FBS), and differentiated by adding 50 μg·mL^−1^ ascorbic acid, 10 mmol·L^−1^ β-glycerophosphate, and 0.1 μmol·L^−1^ dexamethasone. ATDC5 cells were obtained from the European Collection of Authenticated Cell Cultures (ECACC) and cultured in a 1:1 mixture of Dulbecco’s modified Eagle medium (DMEM) and Ham’s F12 medium supplemented with 5% FBS, 1% insulin, transferrin and selenium (Thermo Fisher). ATDC5 cells were differentiated in the presence of 10 mmol·L^−1^ β-glycerophosphate (Sigma) and 50 μg·mL^−1^ 2-phospho-L-ascorbic acid (Sigma). MC3T3-E1 cells were transfected with pmirGLO Dual-Luciferase (Promega) alone or in combination with control or miR-29a-3p mimic (mirVana mimics; Thermo Fisher Scientific) using Lipofectamine 3000 (Thermo Fisher Scientific). MC3T3-E1 cells were transfected with control or miR-29a-3p mimic alone or with control or miR-29a-3p inhibitor (mirVana inhibitors; Thermo Fisher Scientific) using Lipofectamine RNAiMAX (Thermo Fisher Scientific). Primary monocytes were isolated from bone marrow using the EasySep Mouse Monocytes Isolation Kit (StemCell Technologies). Monocytes were differentiated into macrophages in DMEM supplemented with 10% FBS and 15% L929 cell supernatant.

### 3′ RACE-PCR

cDNA was prepared from ≤1 μg RNA using SuperScript IV Reverse Transcriptase (Thermo Fisher Scientific) and the Universal Adapter primer (Table S14). cDNA was amplified by PCR using a gene-specific forward primer (Table S14), the Universal Amplification Reverse primer (Table S14), and the proofreading, thermostable Platinum SuperFi II DNA Polymerase (Thermo Fisher Scientific). The resultant blunt-end PCR product was cloned into the Zero Blunt^®^ TOPO^®^ vector using the Zero Blunt^®^ TOPO^®^ PCR Cloning Kit (Thermo Fisher Scientific). Clones were then sequenced using Sanger DNA Sequencing (GENEWIZ).

### Luciferase assay and construction of pmirGLO Dual Luciferase reporters

Different isoforms and regions of the Col1a1 and Col1a2 3′ UTRs were cloned into the pmirGLO Dual Luciferase vector (Promega) downstream of the FLuc CDS as follows. The pmirGLO Dual Luciferase vector was linearized using the restriction enzyme PmeI (NEB). Each 3′ UTR/isoform was amplified from cDNA using the Platinum SuperFi II DNA Polymerase (Thermo Fisher Scientific) and the In-Fusion primer pairs, whose sequences are listed in Table S14. The resultant PCR product was then cloned into the linearized pmirGLO Dual Luciferase vector using the In-Fusion HD Cloning Kit (Takara). Luciferase assays were performed using the Dual-Glo^®^ Luciferase Assay System (Promega) according to the manufacturer’s instructions.

### Labeling of nascent RNA and analysis of RNA decay

We used the Click-iT Nascent RNA Capture Kit (Thermo Fisher Scientific) according to the manufacturer’s instructions. Briefly, cells were incubated with EU-containing medium for 2 h (pulse step). The cells were then divided into two groups: the first group was harvested to determine the total level of EU-labeled RNA, while the second group was incubated in EU-free medium for 24 h (chase step). In each group, total RNA was isolated from the harvested cells and subjected to a copper-catalyzed click reaction with azide-modified biotin. EU-labeled RNA was then captured using streptavidin magnetic beads and used for cDNA preparation as described above. The levels of total mRNA and the lAPA isoform were quantified by qPCR as described above. The % EU-labeled mRNA that remained (Fig. 4g) was calculated by normalizing the level of EU-labeled mRNA that was measured after the 24-h chase (measured in the second group) to the total level of EU-labeled mRNA that was synthesized during the 2-h pulse (measured in the first group).

### Statistical analysis

The K–S test was used to compare distributions between gene sets. For the qPCR and luciferase assays, the unpaired Student’s t test was used to determine statistical significance between two groups, whereas ANOVA (followed by Tukey’s post hoc test) was used to determine statistical significance among three or more groups. Statistical analyses were performed using GraphPad Prism software. The following symbols were used to indicate significance: (ns) *P* ≥ 0.05; *P < 0.05; **P < 0.01; ***P < 0.001; ****P < 0.000 1.

## Supplementary information

Revised Supplementary Figures (S1-S14)

Revised Supplementary Figure Legends

Elbarbary-Table S1

Elbarbary-Table S2

Elbarbary-Table S3

Elbarbary-Table S4

Elbarbary-Table S5

Elbarbary-Table S6

Elbarbary-Table S7

Elbarbary-Table S8

Elbarbary-Table S9

Elbarbary-Table S10

Elbarbary-Table S11

Elbarbary-Table S12

Elbarbary-Table S13

Elbarbary-Table S14

## Data Availability

Raw RNA-seq data have been deposited in NCBI’s Gene Expression Omnibus^49^ and are accessible with the GEO Series accession number GSE205053.
