# Supplementary material for: Transcript shortening via alternative polyadenylation promotes gene expression during fracture healing
Source: Bone Res. 2023 Jan 3;11:5. doi: 10.1038/s41413-022-00236-7 (PMC9810729; doi:10.1038/s41413-022-00236-7)
Supplement: Supplementary file 1 — Revised Supplementary Figures (S1-S14) [file 41413_2022_236_MOESM1_ESM.pdf]

Fig. S1

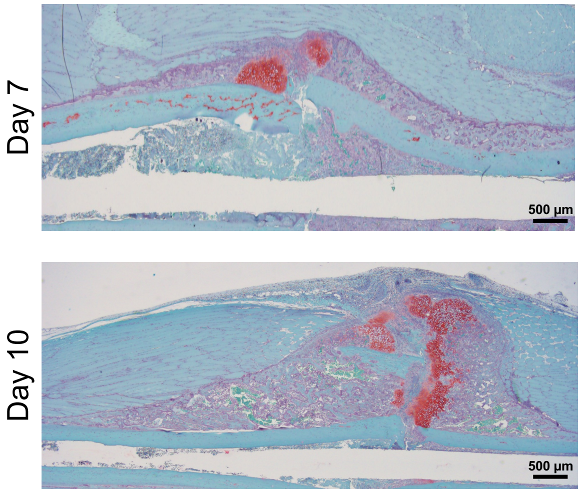

Fig. S2

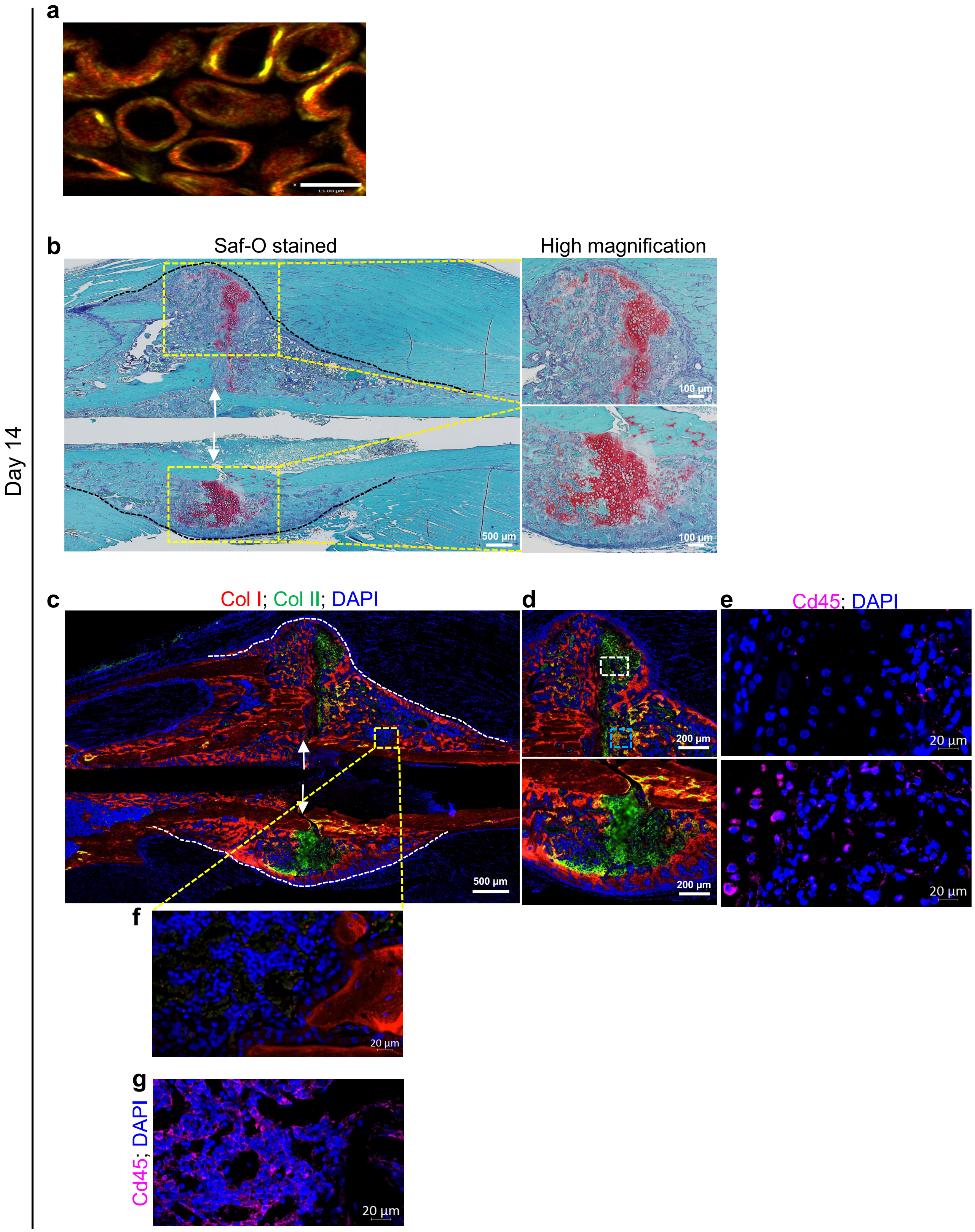

Day 14
a
13.00 µm
b
Saf-O stained
High magnification
100 µm
100 µm
500 µm
c
Col I; Col II; DAPI
500 µm
d
200 µm
200 µm
e
Cd45; DAPI
20 µm
20 µm
f
20 µm
g
Cd45; DAPI
20 µm

Fig. S3

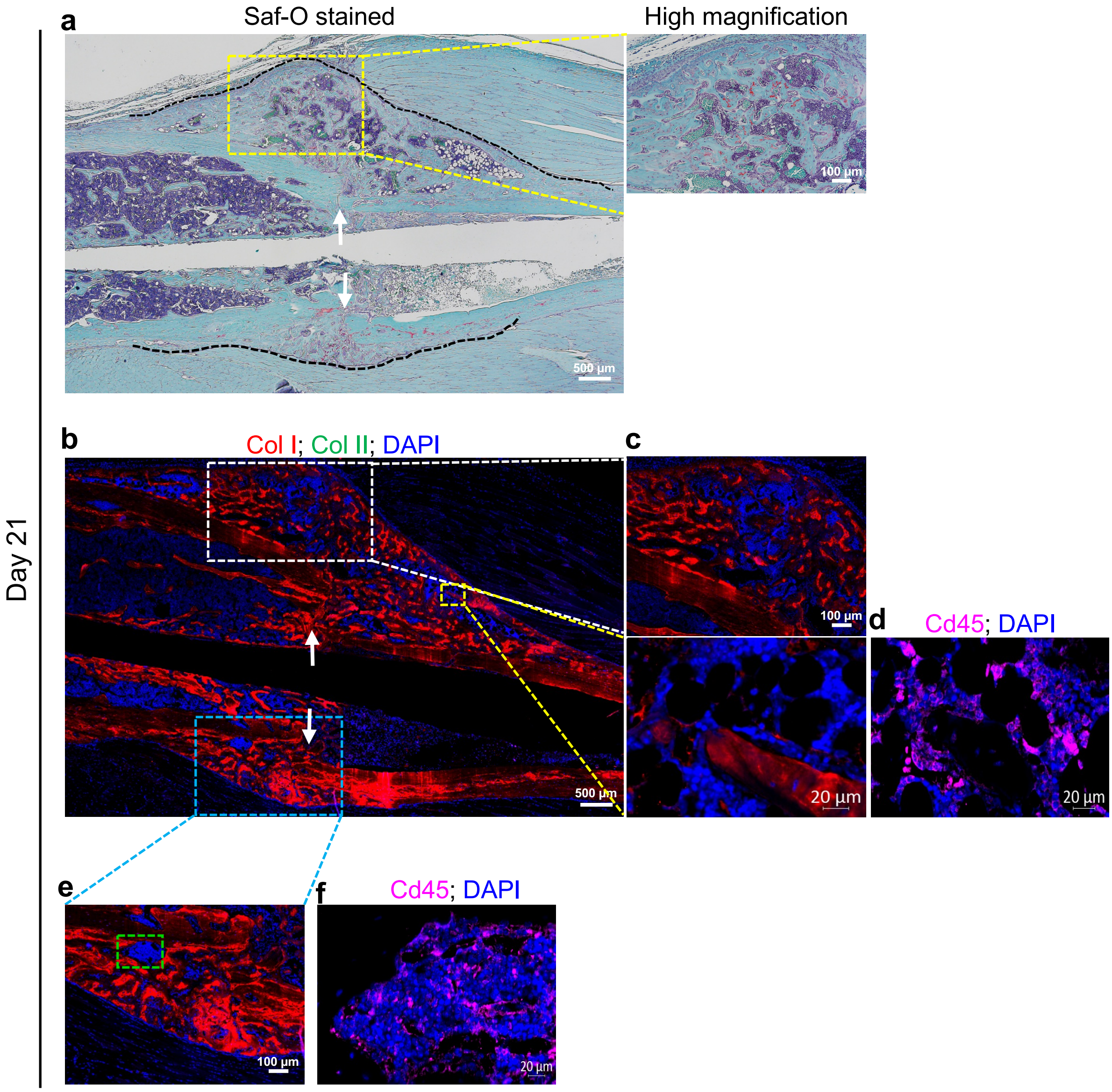

a
Saf-O stained
High magnification
100 µm
500 µm
Day 21
b
Col I; Col II; DAPI
c
100 µm
500 µm
20 µm
d
Cd45; DAPI
20 µm
e
100 µm
f
Cd45; DAPI
20 µm

Fig. S4

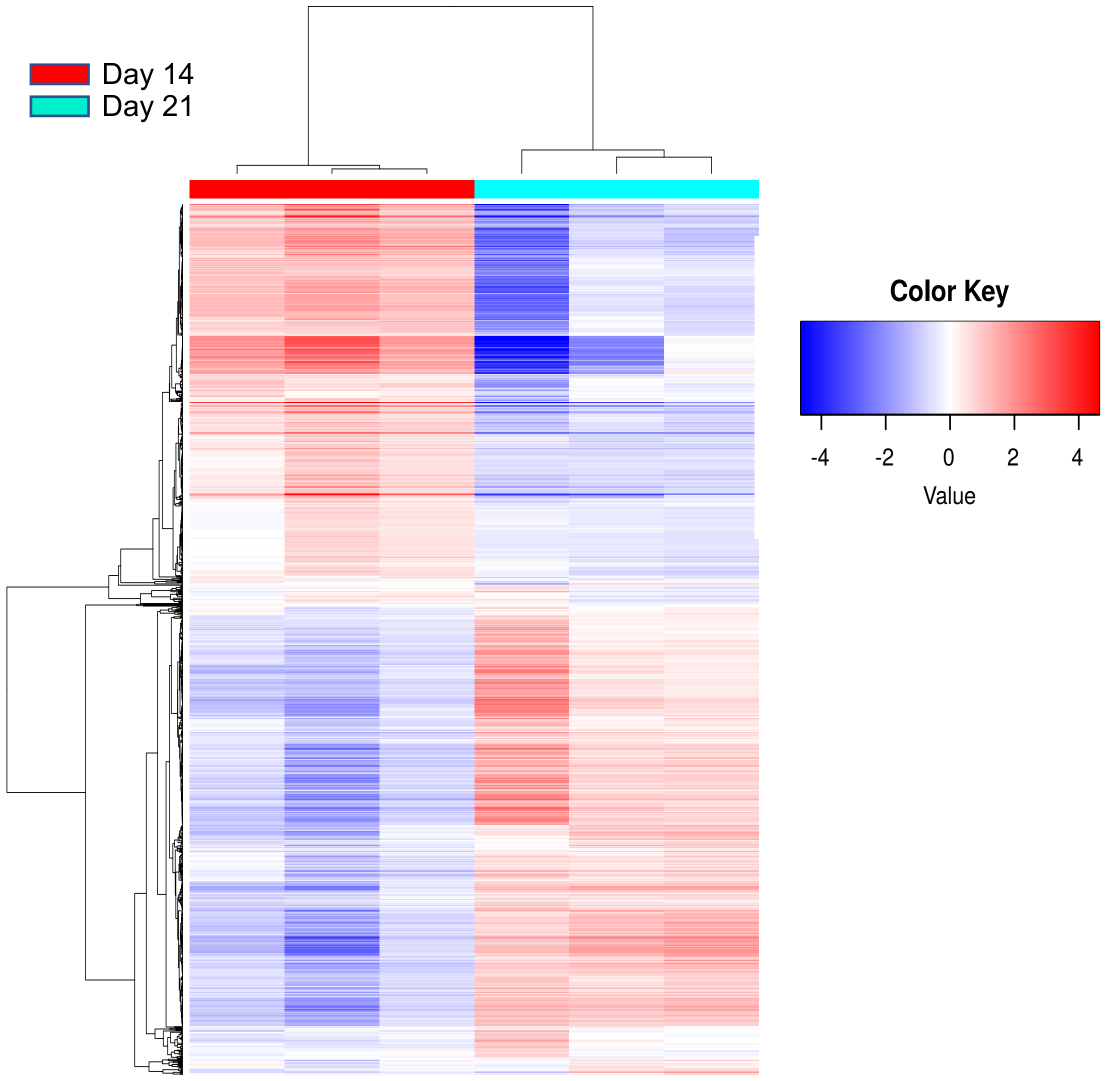

Fig. S5

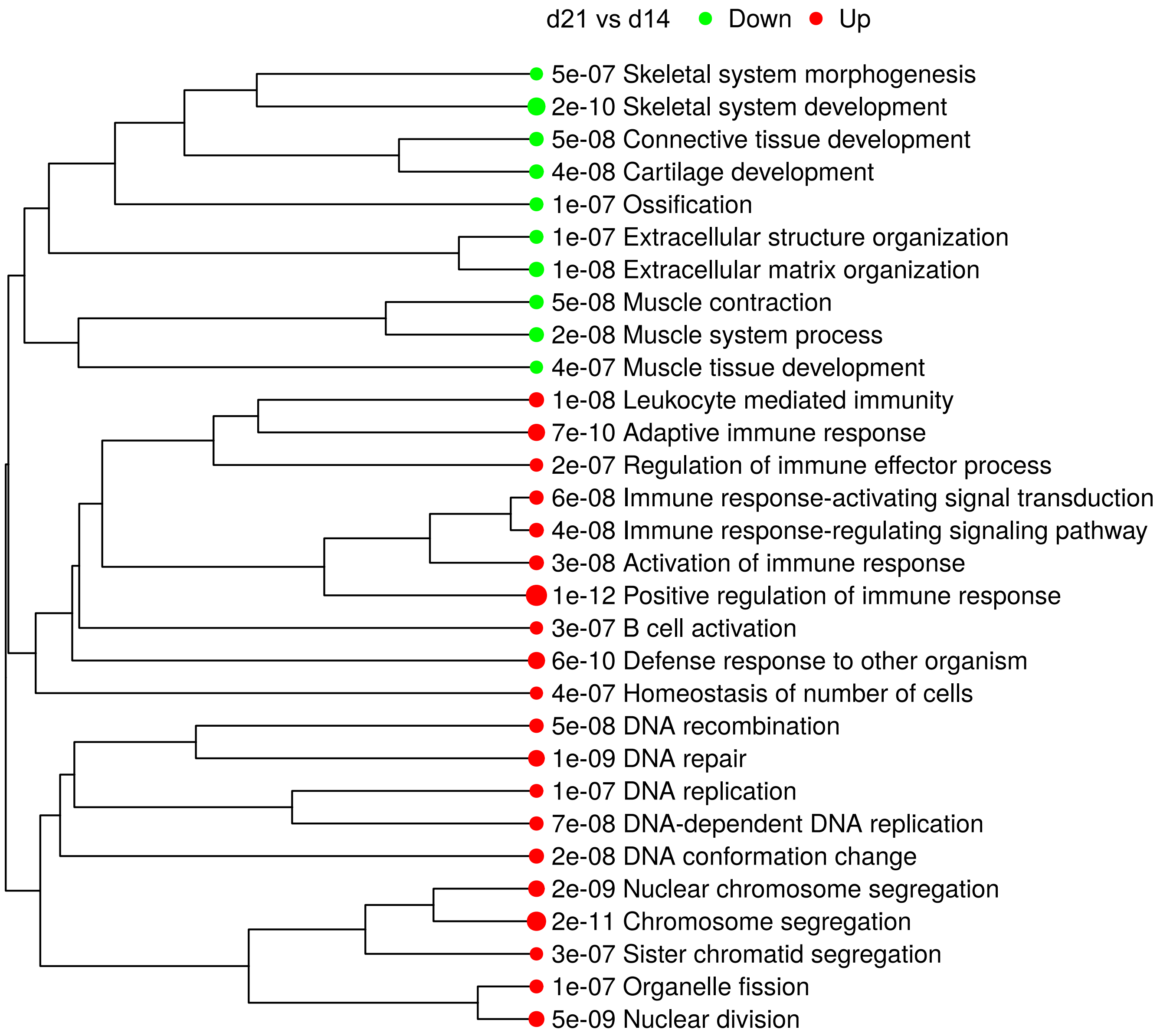
d21 vs d14 Down Up
5e-07 Skeletal system morphogenesis
2e-10 Skeletal system development
5e-08 Connective tissue development
4e-08 Cartilage development
1e-07 Ossification
1e-07 Extracellular structure organization
1e-08 Extracellular matrix organization
5e-08 Muscle contraction
2e-08 Muscle system process
4e-07 Muscle tissue development
1e-08 Leukocyte mediated immunity
7e-10 Adaptive immune response
2e-07 Regulation of immune effector process
6e-08 Immune response-activating signal transduction
4e-08 Immune response-regulating signaling pathway
3e-08 Activation of immune response
1e-12 Positive regulation of immune response
3e-07 B cell activation
6e-10 Defense response to other organism
4e-07 Homeostasis of number of cells
5e-08 DNA recombination
1e-09 DNA repair
1e-07 DNA replication
7e-08 DNA-dependent DNA replication
2e-08 DNA conformation change
2e-09 Nuclear chromosome segregation
2e-11 Chromosome segregation
3e-07 Sister chromatid segregation
1e-07 Organelle fission
5e-09 Nuclear division

Fig. S6

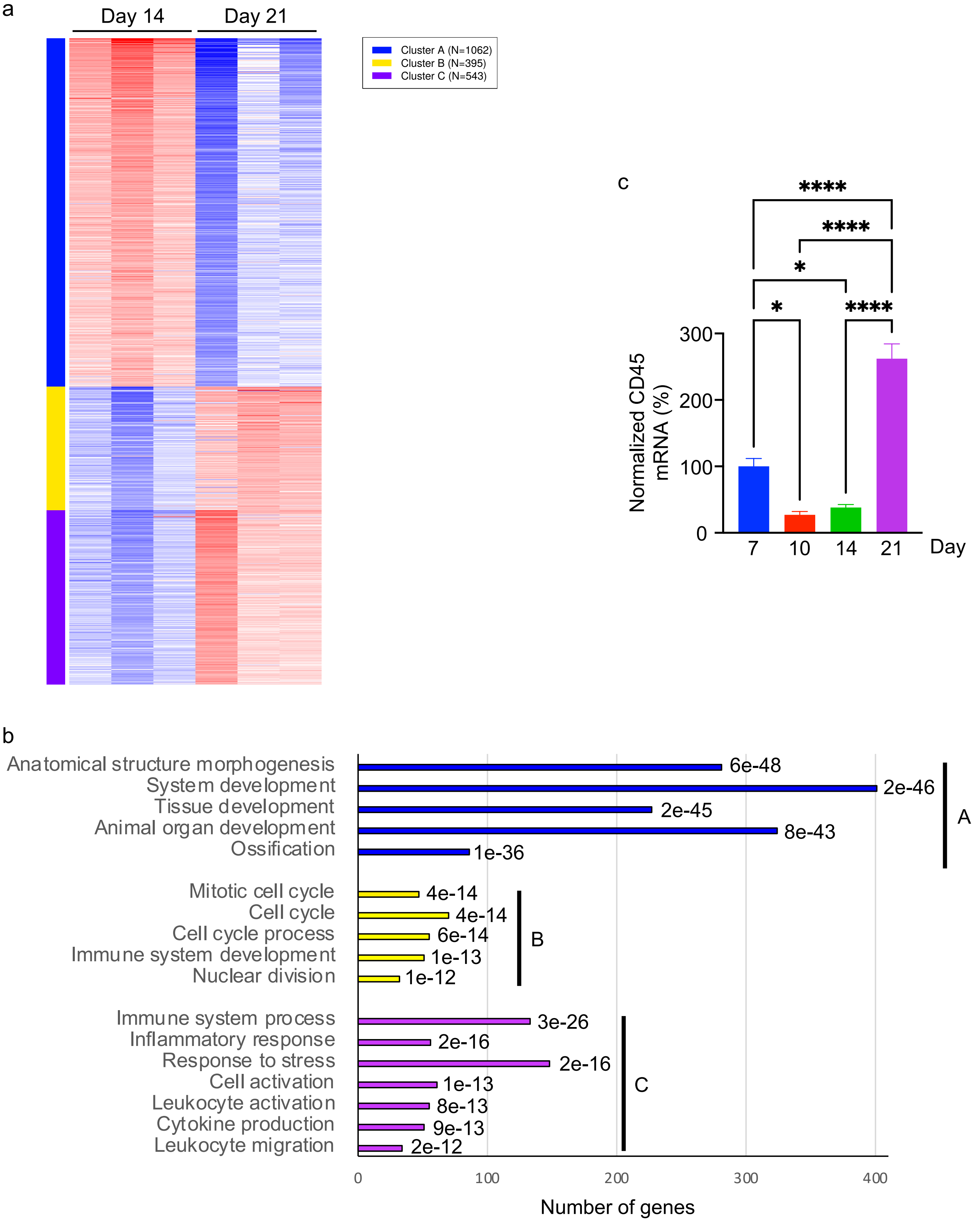
a
Day 14
Day 21
Cluster A (N=1062)
Cluster B (N=395)
Cluster C (N=543)
b
Anatomical structure morphogenesis
System development
Tissue development
Animal organ development
Ossification
Mitotic cell cycle
Cell cycle
Cell cycle process
Immune system development
Nuclear division
Immune system process
Inflammatory response
Response to stress
Cell activation
Leukocyte activation
Cytokine production
Leukocyte migration
6e-48
2e-46
2e-45
8e-43
1e-36
4e-14
4e-14
6e-14
1e-13
1e-12
3e-26
2e-16
2e-16
1e-13
8e-13
9e-13
2e-12
A
B
C
0
100
200
300
400
Number of genes
c
****
****
*
*
****
300
200
100
0
Normalized CD45 mRNA (%)
7
10
14
21
Day

Fig. S7

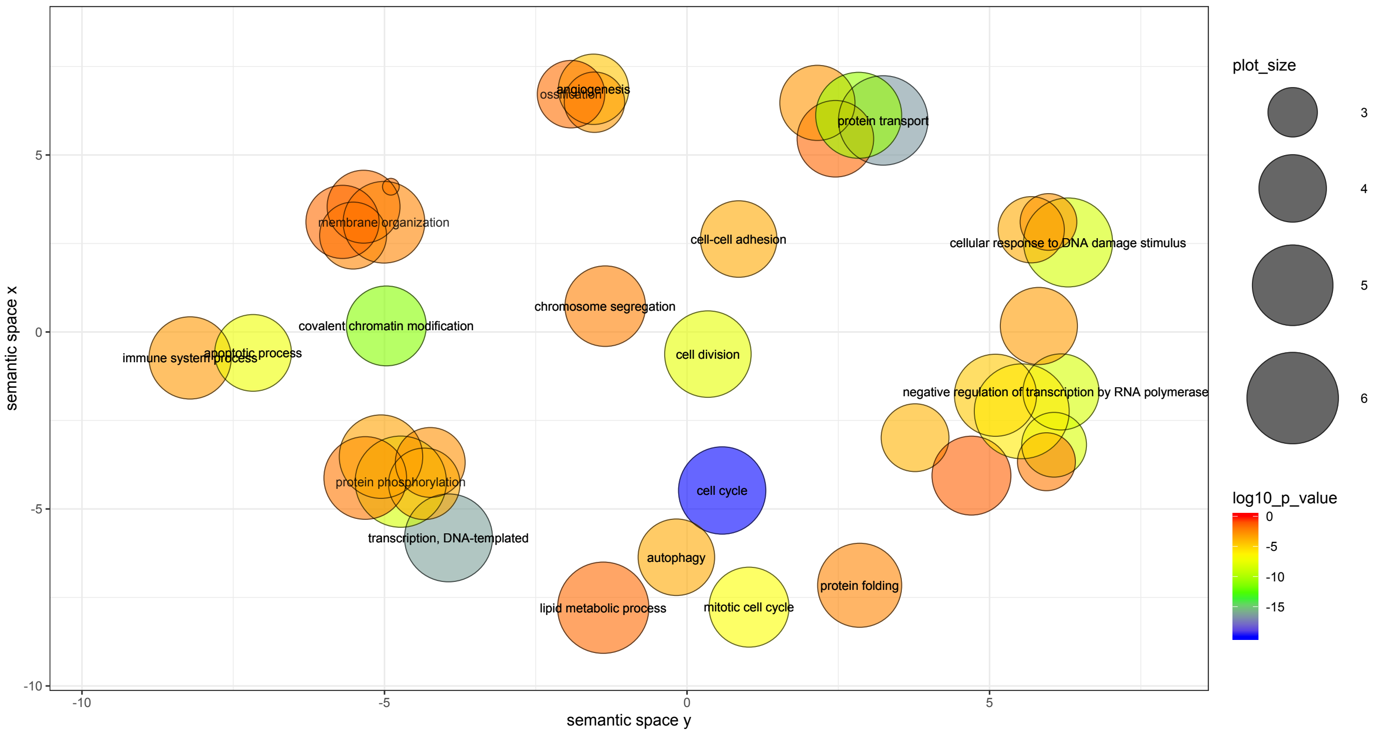

ossification
angiogenesis
protein transport
membrane organization
cell-cell adhesion
cellular response to DNA damage stimulus
chromosome segregation
covalent chromatin modification
immune system process
apoptotic process
cell division
negative regulation of transcription by RNA polymerase
protein phosphorylation
cell cycle
transcription, DNA-templated
autophagy
protein folding
lipid metabolic process
mitotic cell cycle
semantic space x
semantic space y
plot_size
3
4
5
6
log10_p_value
0
-5
-10
-15

a

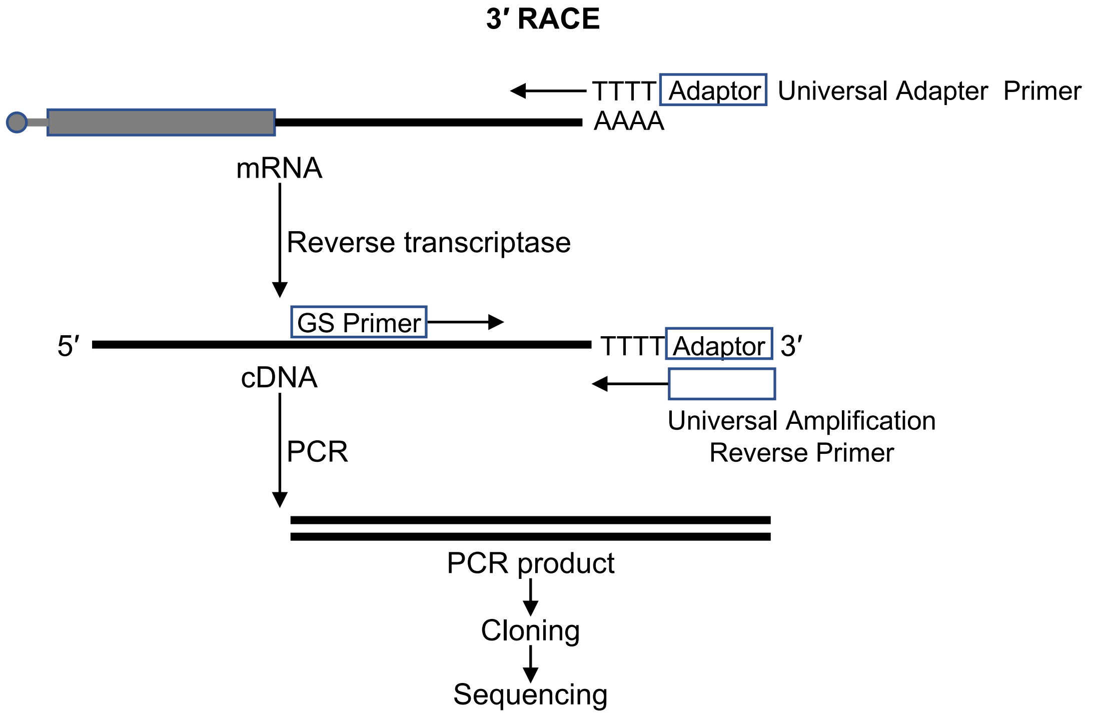
3′ RACE
TTTT
Adaptor
Universal Adapter Primer
AAAA
mRNA
Reverse transcriptase
GS Primer
5′
TTTT
Adaptor
3′
cDNA
Universal Amplification Reverse Primer
PCR
PCR product
Cloning
Sequencing

b

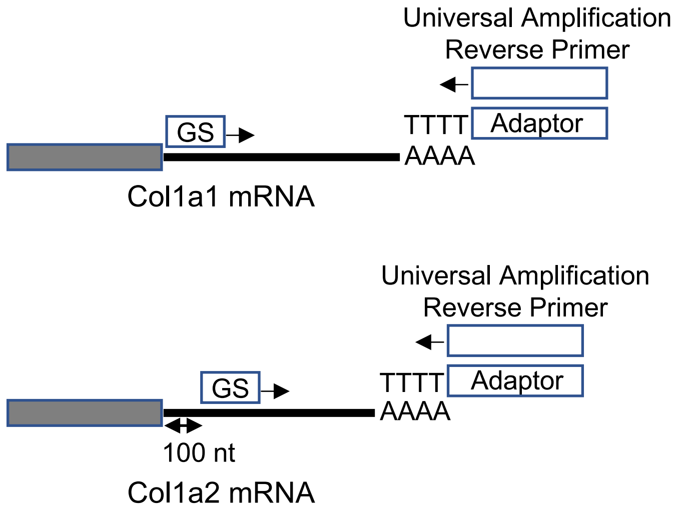
Universal Amplification Reverse Primer
TTTT
Adaptor
GS
AAAA
Col1a1 mRNA
Universal Amplification Reverse Primer
TTTT
Adaptor
GS
AAAA
100 nt
Col1a2 mRNA

a

Col1a1 cUTR and aUTR

ACTCCCTCCACCCCAATCTGGTTCCCTCCCACCCAGCCCACTTTTCCCCAACCCTGGAAACA
GACGAACAACCCAAACTCAATTTCCCCCAAAAGCCAAAAAATATGGGAGATAATTTCACATGGA
CTTTGGAAAACATTTTTTTTCCTTTGCATTCACCTTTCAAACTTAGTTTTTACCTTTGACCAACT
GAACGTGACCAAAAACCAAAAGTGCATTCAACCTTACC***AAAAAAGAAAAAAAAAAAAAAGAA***
TAAATAAATAACTTTTTAAAAAAGGAAGCTTGGTCCTCTTGCTTGAAGACCTATGTGGGTATA
AGTCCCTTCCTGCCCACTTGGCTTATGATACCCTAATGCTGCCTTTTCTGCTCCTTTCTCCAC
CCCCTCTTGGGGCCTCTCCTCCATTGCTCCCCAAATTTAAGTCTCCCCCAAGACACAGGAAA
TAATGCATTGTCTGCCCAGCAAACAAAGGCAATGCTGAAATGTCCCACCAGCCCCTCAACCC
CGTCTACTTCCCTACCCAGCACCCTCAAATCCTGCTGGGACATGGGGTTCTTGGACTGTTGA
AGGAACCTAACCATCTGGCATCTCCATGGCCTCTGCAACAAACCCCCCACTTTTTTCTCTCCC
CCCCCCCCCAGGGAGGGCCTGTGCTTTGGGCAGCCACCTGCCCCTCTCAGGGGTTTGGAG
CCAGGCAGGGTCACAGCAGACTGGAAACATCGGACATGCATGTGCAGGCTGGGTGGGAGAG
ACCGTTCTATTCCTCAGTGCAATTGTGTTGCTGAAAGACTACCTCGTTCTTGTCTTTGTGTGTC
ACCGGGGCAACTGTGTGGGGGCGGGGATGGGGGCAGGGTGGCAGCACGCCCAGTTTGGTA
TCAAAGGTGCTACATCTCTGTGAAGGGGTGGGGTGGGAAGGAATTTCTGGTGCTATAGAAGC
TGAGATGCTCCCTAGACCAGCAAATGTTTCTTTTGTTCAAAGTATTTTTTATTCTTTTTTTTTTTT
TTTTTAATGGATAGGGACTTGTGTGAATTGTTGGGGTTTTTTTTTTTTTTTTTTTTGGTTTTGTTT
TTTTTTGTTTTGTTTTGTTTTTTTTTCCTGAAGGTGCTATTTAACAAGGGAGAAGAGAGTGCGGG
GACTTCACCCTGCCCACTCTCTACTCTCTCTCCACTCTTCTAGTTCCTGGGCCTATCTGATCT
CTCTCTTTCTTCTGAAACCCTCCCCTCTTGCTGCTGCTCCCTCCCCTCTGCCTCTCTCTTGGT
CTGTCCTGCATCAGGGTTTCAGAGCACCACTTTCCAAAGCACAAAACAGTTTTTACCCCTGGG
CTGGGAGGAAACAAGAGACTCTGTACCTATTTTGTATGTGTATAATAATTTGAGATGTTTTTAA
TTATTTTGATTGCTGG*AATAAA*GCATGTGGAAATGACCC

b

Col1a2 cUTR and aUTR

GTGAACTCAACCTAAATTAAAAACCAAAAACCCCTGAAAAAACTTTCTCTTTGCCGTTTCCTCC
TTTAAAAAAAAAAACAAAAAACAAAAACTGAAAGCTGAATCCTTCCATGTCTTCTATGCATCTA
CATCTTAAACTGTGGGCAAAAGAGAAGGATTGGTCAGAGCAGTGTGCAATATGATCCAACTA
AGTCTCCTCCCTTGGCCCCTCCCCAAAATGTTTGCAGTGTTATTTTTGTGGGTTTTTTTTTAAC
ACCCTGACACCTGTTGTGGACATTGTCAACCTTTGTAAGAAAACCC***AAATAAAAATTGAAAA***
ATAAAATAAAAAGAAACCCATGAACATTCGCACCACTTGTGGCTTCTGACTATCTTCCACAG
AGGGAAGTTTAAAACCCAAACTTCCAAAGGTTTGAACTACCTCAAGACACTTTGCAGTGAGTG
TAGACCATCCCAATGAGAGTTGACCAAGGCTGACACGAACTGAGGTACTTGTTTTGTTTTGCT
TGCAACACAAAGGTGCTAATTAGTAGTATTTCAGATACTTGAAGAATATGAACGGTGCTGGAA
GAATTACAGAAGAAACAATCCTCCACATTGATGTGCATTGTGTGTGATTTTTAAATTTGACTTA
GCAGTCCTCTTTCTCATCTTATCCCCAACTACAGGAATGCAGGCCACTTGCCCCAGGTTCTCT
CTTCATTAGATTCAGCATTCATTCTTTAACAGCCTCATTTTCCTTCTTGCCCATGGTTCCAAAG
AAGTCTTGTTTCTTGGACAAGCAGAAAAATTGAATTGTACCTATTTTGTATATGTGAGATGTTT
AAATAAATTGTGAAAAATAAAATAAAGCATTTTTGGTTTTCCAAAAGAAAATATTGAGT

Fig. S10

a

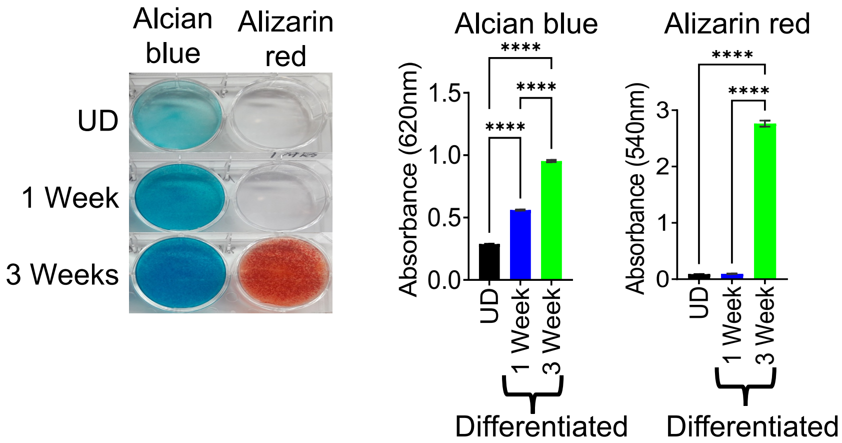
Alcian blue
Alizarin red
UD
1 Week
3 Weeks
Alcian blue
Absorbance (620nm)
0.0
0.5
1.0
1.5
****
****
****
UD
1 Week
3 Week
Differentiated
Alizarin red
Absorbance (540nm)
0
1
2
3
****
****
UD
1 Week
3 Week
Differentiated

b

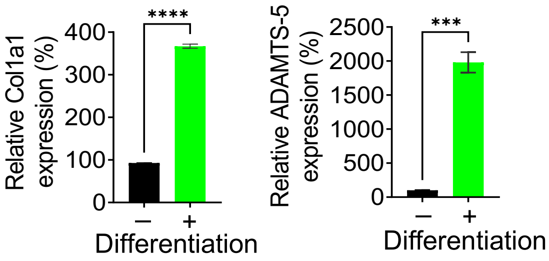
Relative Col1a1 expression (%)
0
100
200
300
400
****
−
+
Differentiation
Relative ADAMTS-5 expression (%)
0
500
1000
1500
2000
2500
***
−
+
Differentiation

a **Fig. S11**

MREs of miR-29a-3p in the 3' UTR of mouse Col1a1 mRNA

1 actccctcca ccccaatctg gttccctccc acccagccca cttttcccca accctggaaa

61 cagacgaaca acccaaactc aatttccccc aaaagccaaa aatatgggag ataatttcac

121 atggactttg gaaaacattt tttttccttt gcattcacct ttcaaactta gtttttacct

181 ttgaccaact gaacgtgacc aaaaaccaaa agtgcattca accttaccaa aaaagaaaaa

241 aaaaaaaaga ataaataaat aacttttaa aaaaggaagc ttggtcctct tgcttgaaga

301 cctatgtggg tataagtccc ttcctgccca cttggcttat gataccctaa tgctgccttt

361 tctgctcctt tctccacccc ctcttggggc ctctcctcca ttgctcccca aatttaagtc

421 tcccccaaga cacaggaaat aatgcattgt ctgcccagca aacaaaggca atgctgaaat

481 gtcccaccag cccctcaacc ccgtctactt ccctacccag caccctcaaa tcctgctggg

541 acatggggtt cttggactgt tgaaggaacc taaccatctg gcatctccat ggcctctgca

601 acaaacccc cactttttc tctccccccc cccccaggga gggcctgtgc tttgggcagc

661 cacctgcccc tctcaggggt ttggagccag gcagggtcac agcagactgg aaacatcgga

721 catgcatgtg caggctgggt gggagagacc gttctattcc tcagtgcaat tgtgttgctg

781 aaagactacc tcgttcttgt ctttgtgtgt caccggggca actgtgtggg ggcggggatg

841 ggggcagggt ggcagcacgc ccagtttggt atcaaa**ggtg cta**catctct gtgaaggggt

901 ggggtgggaa ggaatttc**tg gtgcta**taga agctgagatg ctccctagac cagcaaatgt

961 ttcttttgtt caaagtattt tttattcttt tttttttttt ttttaatgga tagggacttg

1021 tgtgaattgt tggggttttt tttttttttt tttttggttt tgtttttttt tgttttgttt

1081 tgtttttttt cctgaa**ggtg cta**tttaaca agggagaaga gagtgcgggg acttcaccct

1141 gcccactctc tactctctct ccactcttct agttcctggg cctatctgat ctctctcttt

1201 cttctgaaac cctcccctct tgctgctgct ccctcccctc tgcctctctc ttggtctgtc

1261 ctgcatcagg gtttcagagc accactttcc aaagcacaaa acagttttta cccctgggct

1321 gggaggaaac aagagactct gtacctattt tgtatgtgta taataatttg agatgttttt

1381 aattattttg attgctggaa taaagcatgt ggaaatgacc caaaaaaaaa aaaaaaaaa

b

MREs of miR-29a-3p in the 3' UTR of mouse Col1a2 mRNA

1 gtgaactcaa cctaaattaa aaaccaaaaa cccctgaaaa aactttctct ttgccgtttc

061 ctcctttaaa aaaaaaaaca aaaaacaaaa actgaaagct gaatccttcc atgtcttcta

121 tgcatctaca tcttaaactg tgggcaaaag agaaggattg gtcagagcag tgtgcaatat

181 gatccaacta agtctcctcc cttggcccct ccccaaaatg tttgcagtgt tatttttgtg

241 ggtttttttt taacaccctg acacctgttg tggacattgt caacctttgt aagaaaaccc

301 aaataaaaat tgaaaaataa aataaaaaga aacccatgaa cattcgcacc acttgtggct

361 tctgactatc ttccacagag ggaagtttaa aacccaaact tccaaaggtt tgaactacct

421 caagacactt tgcagtgagt gtagaccatc ccaatgagag ttgaccaagg ctgacacgaa

481 ctgaggtact tgttttgttt tgcttgcaac acaaa**ggtgc ta**attagtag tatttcagat

541 acttgaagaa tatgaacggt gctggaagaa ttacagaaga aacaatcctc cacattgatg

601 tgcattgtgt gtgattttta aatttgactt agcagtcctc tttctcatct tatccccaac

661 tacaggaatg caggccactt gccccaggtt ctctcttcat tagattcagc attcattctt

721 taacagcctc attttccttc ttgcccatgg ttccaaagaa gtcttgtttc ttggacaagc

781 agaaaaattg aattgtacct attttgtata tgtgagatgt ttaaataaat tgtgaaaaat

841 aaaataaagc atttttggtt ttccaaaaga aaatattgag t

a

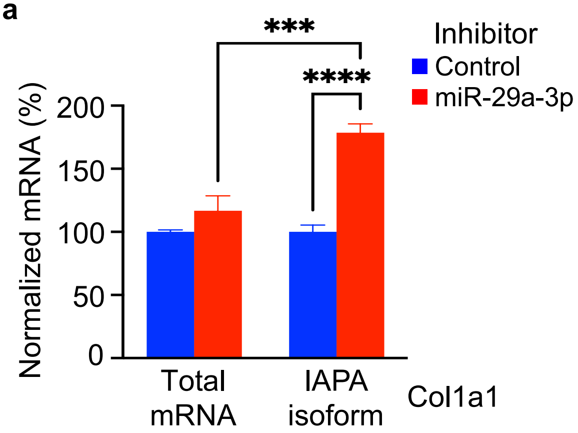
Inhibitor
Control
miR-29a-3p
***
****
Normalized mRNA (%)
0
50
100
150
200
Total mRNA
IAPA isoform
Col1a1

b

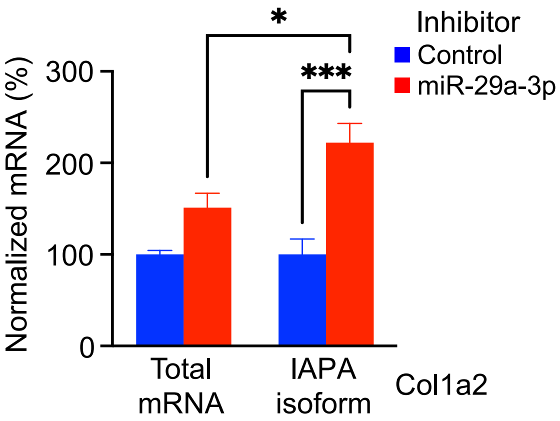
Inhibitor
Control
miR-29a-3p
*
***
Normalized mRNA (%)
0
100
200
300
Total mRNA
IAPA isoform
Col1a2

Fig. S13

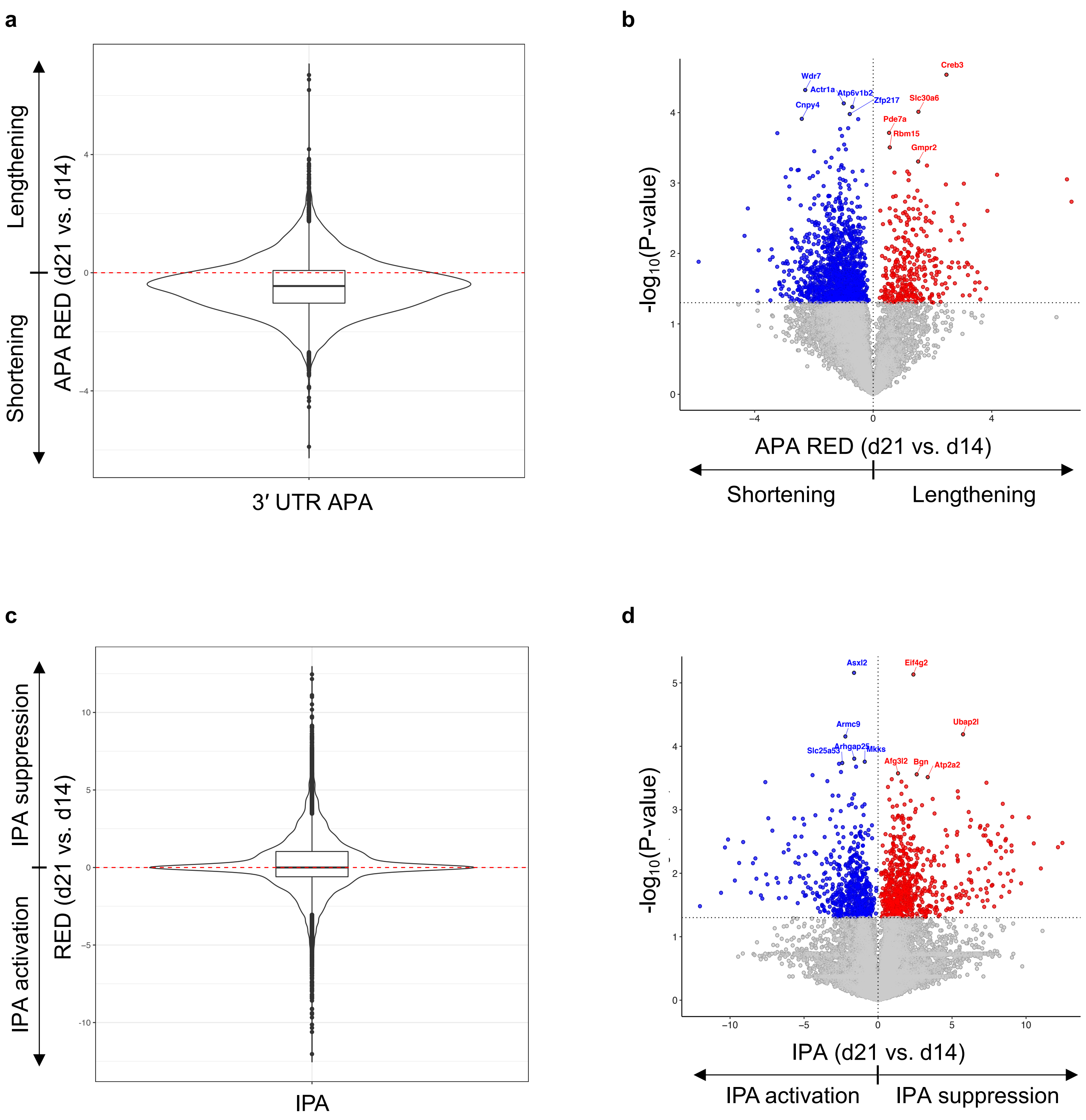
a
Lengthening
Shortening
APA RED (d21 vs. d14)
3′ UTR APA
b
-log10(P-value)
APA RED (d21 vs. d14)
Shortening
Lengthening
Wdr7
Actr1a
Atp6v1b2
Zfp217
Cnpy4
Creb3
Slc30a6
Pde7a
Rbm15
Gmpr2
c
IPA suppression
IPA activation
RED (d21 vs. d14)
IPA
d
-log10(P-value)
IPA (d21 vs. d14)
IPA activation
IPA suppression
Asxl2
Eif4g2
Armc9
Ubap2l
Slc25a53
Arhgap25
Mkks
Afg3l2
Bgn
Atp2a2

Fig. S14

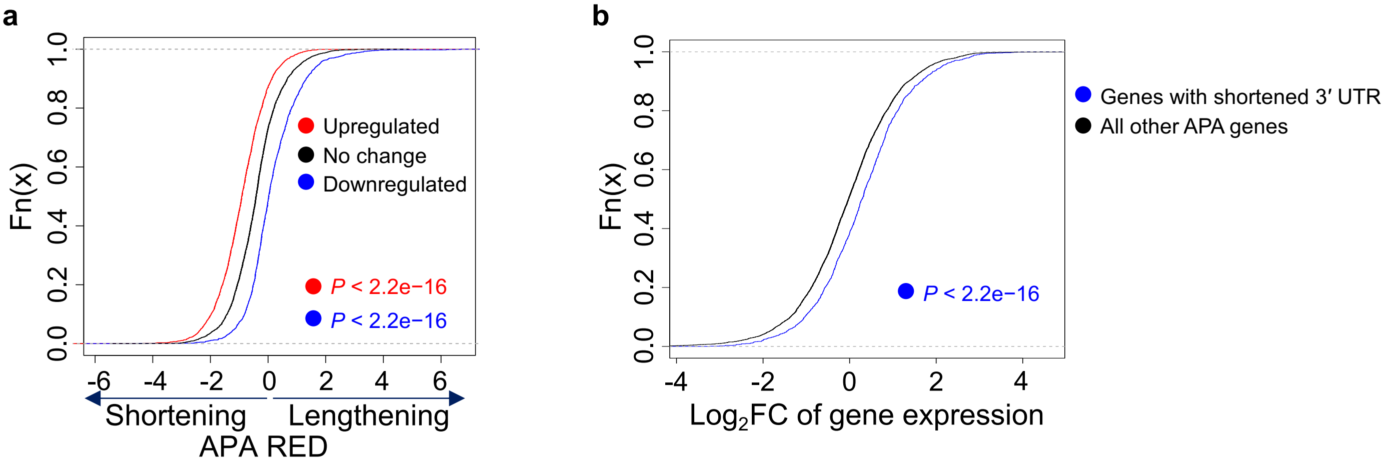

c

Irak2 cUTR and aUTR

TGACCAGCGCACAGCTGAAGACCCTTATTCTCAACTGGACAGACAAACGTCCAGGCAGGGAAGA
GGTCTTTGGCTGCCCAAGGTCTGTTGAGACCCACACAACCCAAACATCAGCCTGAACAGAAGGA
AACATGGCTTCATCAGAGTCGAGTTGCAGGGGGGTGGGGGTAGGGGACCTCAGCTTATACAGA
CACCATATCCAGGAAGCACTCCTCCTCTCTGAGTCGTCTTAGTTAGAGGGCTGTGAAGTGGAAC
ACCTTGTATGTGATCCAAGGATTTGGGTGGTTTTTGTGGTTCCGGGGAACCCACGTTGTTGACCA
CCCCAAGAGATGCTGTTGCCCCCAAACCTCTTAGTTCTCCACAACGGACAGAGCAATCCTGCAG
CATCCGTGGCGTTTTCCAGAGTTATCATCTGTCAGTCATATTTGTAGACAGCTTTCTGCAAATTCC
CACTTCTTAAAAGACCTACAGCCGGTGTCTCCGCAAGTTCTCAAGTTCAGGACTGCAAAAGCAG
GACGGTTGGCAAAGGACTCCGTAGGCTTTCTCCTTCTTCAGTTGGTGATCCTGTAAGCCACCGG
TATTTAGGAACTGCCTGCTTTTCACCTTCGACCTCCCATTGTGCCCATATTTATTTTAATGGAGAC
TCAGAGGTTGTGTGTTATTTATAGCTAGGTGAAGAAGCATGAGAAACAGTCTCATGTTTGATTCT
GCTGCTAGTTAAGTCAATCACTTGGTGGGTGGTAGCTCAGTATCCTTGTAAGATAACCCTGCTCT
GTTCTCCCTGGGGAAGAACAGAGCTGCTCTATTCAGCTCTTGGGAGGATGGGTGCAGCGGCTG
CAGAAGCACTTTGAAAATGTATCTGTTCTGTGTTCTCAATGGAGTAAAAGGTATGTAGTTCATGG
CTCTGCCATGTAGAAAATGTTTATGAAGTGAATATATTTTTGAAAAGTCATGTTGCAAACATGTTTT
GTAAAACAAAGCTATTTCTTTCTTTTTTTTTTTGTTTTTTTTTTGTTTTTTCGAGACAGGGTTTCTCT
GTATAGCCCTGGCTGTCCGGGACCTCACTTTGTAGACCAGGCTGGCCTCGAACTCAGAAATCCG
CCTGCCTCTGCCTCCCGAGTGCTGGGATTAAAGGCGTGCGCCACCATGCCCGGCTAACAAAGC
TATTTCTAATTCACTATTTAAAAAAAGAATTCCATTATAGTGACAAAATAAAAATGTTTGTAATCTA
GCAGCTGTCTTATAAAAAAAAAAAAAAA
