# Supplementary material for: Transcript shortening via alternative polyadenylation promotes gene expression during fracture healing
Source: Bone Res. 2023 Jan 3;11:5. doi: 10.1038/s41413-022-00236-7 (PMC9810729; doi:10.1038/s41413-022-00236-7)
Supplement: Supplementary file 2 — Revised Supplementary Figure Legends [file 41413_2022_236_MOESM2_ESM.docx]

**Supplementary figures legends**

**Figure S1.**

Safranin O/fast green staining of callus tissues harvested at the indicated timepoints. The cartilaginous extracellular matrix of the soft callus is stained reddish orange. The scale bar = 500 μm.

**Figure S2.**

**(a)** A representative second harmonic generation (SHG) microscopy image showing the characteristic morphology of hypertrophic chondrocytes. The image was captured on d14 in the callus areas where chondrocytes express hypertrophy markers (**Fig. 1b-d**). The scale bar = 13 μm. (**b, left**) Safranin O/fast green staining of the d14 callus (same sample shown in Fig. 1a). The cartilaginous extracellular matrix of the soft callus is stained reddish orange. The white arrows point to the fracture line. The dashed line outlines the callus. The scale bar = 500 μm. (**b, right**) Magnified images of the regions indicated by the yellow boxes. The scale bar = 100 μm. (**c**) IF co-staining of Col I (red) and Col II (green) of the same d14-callus sample shown in (b). DAPI Stains nuclei. The white arrows point to the fracture line. The dashed line outlines the callus. The scale bar = 500 μm. The tiled image shows the full callus area in which the magnified image presented in **Fig. 1d** was captured. Col II (green) marks the soft callus that occupies the core of the callus, filling and surrounding the fracture gap. Col I marks woven bone that forms in areas distal to the fracture line. Areas where both Col I and Col II proteins are expressed (yellow) surround mineralizing chondrocytes. Bone marrow cells reside in regions that stain positive for DAPI and negative for Col I in the woven bone area (e.g., the area indicated by the yellow box). (**d**) Magnified images showing Col I and Col II co-staining in the same regions shown in (b, right). The scale bar = 200 μm. (**e**) IF staining of CD45 (magenta), which is a general marker of immune cells. DAPI stains nuclei. The Scale bar = 20 μm. The top image was captured in the region indicated by the white box in (d); the image shows low infiltration of immune cells within the core of the soft callus (green-stained areas in c and d). The bottom image was captured in the region indicated by the blue box in (d). Compared to the top image, there was more infiltration of immune cells in regions where Col I is expressed around terminally hypertrophic chondrocytes (yellow-stained areas in c and d). (**f**) A magnified image of the woven bone area indicated by the yellow box in c; the image shows bone marrow cells interwoven with newly formed bone. The Scale bar = 20 μm. (**g**) IF staining of CD45 (magenta). DAPI stains nuclei. The Scale bar = 20 μm. The image was captured in the same region shown in (f). The image shows that immune cells in the d14 callus reside mainly in the bone marrow. All images are representative of 5 mice.

**Figure S3.**

(**a, left**) Safranin O/fast green staining of the d21 callus. The absence of the reddish orange stain indicates resorption of the soft callus. The white arrows point to the fracture line. The dashed line outlines the callus. The scale bar = 500 μm. The image was captured in the same sample shown in Fig. 1e. (**a, right**) A magnified image of the region indicated by the yellow box. the scale bar = 100 μm. The structure of the newly formed woven bone that fills the whole callus area is obvious. (**b**) IF co-staining of Col I (red) and Col II (green) of the same d21-callus sample shown in (a). DAPI stains nuclei. The scale bar = 500 μm. The tiled image shows the full callus area in which the magnified image shown in **Fig. 1f** was captured. The absence of Col II (green staining) indicates complete resorption of the soft callus. (**c, top**) A magnified image of the region indicated by the white box in (b). The scale bar = 100 μm. The bone structure (red staining) is obvious and the bone marrow population in the DAPI^+^ Col I^-^ area is clear. (**c, bottom)** A magnified image captured in the region indicated by the yellow box in (b). The image shows the bone (red)-bone marrow interwoven structure at high magnification. The scale bar = 20 μm. (**d**) IF staining of CD45 (magenta), which is a general marker of immune cells. DAPI stains nuclei. The scale bar = 20 μm. The image was captured in the same region shown in (c, bottom). The image shows the enrichment of immune (CD45^+^) cells in the bone marrow. (**e**) as in (c, top), except that the image shows the woven bone area indicated by the blue box in the lower callus in (b). (**f**) as in (d) except that the image was captured in the region indicated by the green box in (e). All images are representative of 5 mice.

**Figure S4.**

A heatmap of hierarchically clustered gene expression in the RNA-seq data generated from the d14 and d21 calli (**Table S1**). The heatmap demonstrates high clustering of the 3 biological replicates analyzed at each timepoint.

**Figure S5.**

Tree presentation of the Generally Applicable Gene-set Enrichment (GAGE) analysis of the RNA-seq data (**Table S2**). Significantly enriched downregulated (green) and upregulated (red) pathways on d21 relative to d14 are shown. The *P*_adj_ of enrichment for each pathway is given.

**Figure S6.**

(**a**) A heatmap of K-means clustering of the differentially expressed (*P*_adj_ < 0.05) genes on d21 relative to d14 (**Table S3**). Genes were grouped in 3 clusters, in which genes in Cluster A are downregulated on d21 relative to d14, while genes in Clusters B and C are upregulated on d21 relative to d14. The number of genes in each cluster is given. (**b**) Representative pathways in which genes in Cluster A (blue), B (yellow), or C (purple) are enriched. The *P_adj_* of enrichment for each pathway is given. (c) RT-qPCR quantification of CD45 mRNA at the indicated timepoints. The expression level was normalized to that of β-actin mRNA, and the normalized level on d7 was defined as 100. N = 3. The bar graph presents average ± SEM. (*) P < 0.05; (****) P < 0.0001 using one-way ANOVA.

**Fig. S7.**

A scatterplot visualizing the results of GO analysis of d14 APA events. GO analysis was performed using the Database for Annotation, Visualization, and Integrated Discovery (DAVID), and the summarized, non-redundant GO terms are shown.

**Figure S8.**

(**a**) A Scheme for the general protocol of 3′ RACE. cDNA was prepared using a Universal Adaptor Primer that anneals to the poly(A) tail and adds an adaptor to the 3′ end. The following PCR step was performed using a Universal Amplification Reverse Primer, which anneals to the added adaptor, and a gene-specific (GS) forward primer. The resultant PCR product was cloned and subjected to Sanger sequencing to map the 3′ end. (**b**) Presentation of the 3′ RACE of the Col1a1 and Col1a2 mRNAs. The forward GS primer that was used to amplify the 3′ UTR of the Col1a1 mRNA annealed immediately downstream to the stop codon, while the forward GS primer that was used to amplify the 3′ UTR of the Col1a2 mRNA annealed 100 nt downstream to the stop codon (**Table S14**).

**Figure S9.**

(**a**) Sequence of the full-length (i.e., lAPA) 3′ UTR of the Col1a1 mRNA. The sAPA 3′ UTR is underlined. The 3′ end of the sAPA 3′ UTR was mapped by sequencing the 3′ RACE-PCR products (**Fig. 3b, d)**. The SSR (defined by the RepeatMasker program) is in bold italic. Notably, the red-highlighted cytosine nucleotide was mapped as an alternative 3′ end of the sAPA 3′ UTR in 20% of the sequenced clones. However, this might be due to internal annealing of the Universal Adaptor Primer to the A-rich SSR. Notably, even if the red-highlighted cytosine represents a real CPS, it is a minor one anyway. Accordingly, we defined the major proximal CPS as the 3′ end of the sAPA 3′ UTR. (**b**) As in (a), except that the 3′ UTR of the Col1a2 mRNA is shown. The two red-highlighted nucleotides were defined as proximal CPSs: the thymine nucleotide was identified in 4 out of 10 sequenced clones, whereas the cytosine was identified in 6. We defined the major proximal CPS as the 3′ end of the sAPA 3′ UTR.

**Figure S10.**

(**a**) Images (left) and quantification (right) of Alcian blue and Alizarin red staining of ATDC5 cells before (UD; undifferentiated) and after one week or three weeks of differentiation. Alcian blue staining indicates differentiation to chondrocytes, while Alizarin red staining indicates hypertrophic differentiation and mineralization. (**b**) RT-qPCR quantification of the specified chondrocyte-hypertrophy markers using the RNA purified from undifferentiated (−) or differentiated (+) ATDC5 cells. The expression level of each transcript was normalized to that of β-actin mRNA, and the normalized level in the undifferentiated cells was defined as 100. The results demonstrate high expression of chondrocyte-hypertrophy markers in the differentiated ATDC5 cells; these results complement the RT-qPCR data shown in **Fig. 3h**. N = 3. The bar graphs present average ± SEM. (***) P < 0.001; (****) P < 0.0001 using one-way ANOVA in (a) or unpaired Student’s *t* test in (b).

**Figure S11.**

(**a**) Sequence of the 3′ UTR of the Col1a1 mRNA. The 3 MREs of miR-29a-3p are in blue. The sequence of the sAPA 3′ UTR is underlined. The 3 MREs are in the aUTR. (**b**) As in (a), except that the 3′ UTR of the Col1a2 mRNA is shown.

**Figure S12.**

(**a**) RT-qPCR quantification of either the total mRNA or the lAPA isoform of Col1a1 in MC3T3 cells that were transfected with control (blue) or miR-29a-3p (red) inhibitor. A primer pair that binds within the aUTR region was used to specifically amplify and quantify the lAPA isoform (**Table S14**). The expression level was normalized to that of β-actin mRNA, and the normalized level in cells that were transfected with the control inhibitor was defined as 100. N = 4. (**b**) As in (a), except that the total mRNA and the lAPA isoform of Col1a2 were quantified.

The bar graphs present the average ± SEM. (*) *P* < 0.05; (***) *P* < 0.001; (****) P < 0.0001. Significance of difference was calculated using two-way ANOVA.

**Figure S13.**

(**a**) A combination of a violin plot and a boxplot presenting the significant APA events that were identified by APAlyzer (Table S5). The results indicate predominant shortening events. (**b**) A volcano plot of the APAlyzer results. Each dot represents a gene. The significant lengthening and shortening events are shown in red and blue, respectively. The results indicate that the shortening events are prevalent, which is consistent with the results shown in (a). (**c**) As in (a), except that the IPA events are shown. The results indicate no bias toward activation or suppression of IPA on d21 relative to d14 (the median RED value of IPA events is zero). (**d**) As in (b), except that the IPA events are shown. No trend was observed toward IPA activation or suppression, which is consistent with the results shown in (c).

**Figure S14.**

(**a**) CDF curves of the RED values (generated by APAlyzer) of genes whose expression was significantly upregulated (red line), significantly downregulated (blue line), or unchanged (black line) on d21 relative to d14. A significant change in gene expression was defined as *Padj* < 0.05. All the APAlyzer-identified APA events (**Table S5**) are included in the analysis. *P* values (K-S test) indicating the significance of the difference between the red or blue and black genes are given. The results are highly consistent with the CDF curves generated using the InPAS results (**Fig. 7a**). (**b**) CDF curves comparing the expression of genes that exhibited significant 3′ UTR shortening according to APAlyzer (blue line) and all other genes (black line) (**Table S5**). The *P* value (K-S test) is based on comparing the blue and black genes. The results are highly consistent with those generated using the InPAS data (**Fig. 7b**). (**c**) Sequence of the full-length (i.e., lAPA) 3′ UTR of the Irak2 mRNA. The sAPA 3′ UTR is underlined. The 3′ end of the sAPA 3′ UTR was mapped by sequencing the 3′ RACE-PCR products (**Fig. 7e-h)**. Notably, the two red-highlighted cytosine nucleotides were mapped as alternative 3′ ends of the sAPA 3′ UTR; the upstream one was mapped in 60% of the sequenced clones.
